# HIV pre-exposure prophylaxis and its implementation in the PrEP Impact Trial in England: a pragmatic health technology assessment

**DOI:** 10.1016/S2352-3018(23)00256-4

**Published:** 2023-12-01

**Authors:** Ann K Sullivan, John Saunders, Monica Desai, Andrea Cartier, Holly D Mitchell, Sajjida Jaffer, Dana Ogaz, Chiara Chiavenna, Andre Charlett, Victor Diamente, Rainer Golombek, Kaveh Manavi, Cecilia Priestley, Laura J Waters, Ana Milinkovic, Alan McOwan, Claudia Estcourt, Caroline A Sabin, Alison Rodger, Deborah Gold, Brian G Gazzard, Sheena McCormack, O Noel Gill

**Affiliations:** Chelsea and Westminster Hospital National Health Service (NHS) Foundation Trust, London, UK; UK Health Security Agency, London, UK; UK Health Security Agency, London, UK; University College London, London, UK; National Institute for Health and Care Research Health Protection Research Unit (NIHR HPRU) in Blood Borne and Sexually Transmitted Infections at University College London, London, UK; UK Health Security Agency, London, UK; Chelsea and Westminster Hospital National Health Service (NHS) Foundation Trust, London, UK; UK Health Security Agency, London, UK; Chelsea and Westminster Hospital National Health Service (NHS) Foundation Trust, London, UK; UK Health Security Agency, London, UK; UK Health Security Agency, London, UK; UK Health Security Agency, London, UK; Chelsea and Westminster Hospital National Health Service (NHS) Foundation Trust, London, UK; Chelsea and Westminster Hospital National Health Service (NHS) Foundation Trust, London, UK; University Hospitals Birmingham NHS Foundation Trust, Birmingham, UK; Dorset County Hospital NHS Foundation Trust, Dorset, UK; Central and North West London NHS Foundation Trust, London, UK; Chelsea and Westminster Hospital National Health Service (NHS) Foundation Trust, London, UK; Chelsea and Westminster Hospital National Health Service (NHS) Foundation Trust, London, UK; Glasgow Caledonian University, Glasgow, UK; University College London, London, UK; National Institute for Health and Care Research Health Protection Research Unit (NIHR HPRU) in Blood Borne and Sexually Transmitted Infections at University College London, London, UK; University College London, London, UK; National AIDS Trust, London, UK; Chelsea and Westminster Hospital National Health Service (NHS) Foundation Trust, London, UK; Chelsea and Westminster Hospital National Health Service (NHS) Foundation Trust, London, UK; University College London, London, UK; UK Health Security Agency, London, UK

## Abstract

**Background:**

HIV pre-exposure prophylaxis (PrEP) is highly effective in preventing HIV acquisition. To enable routine commissioning of PrEP in England, we aimed to establish population need, duration of need, PrEP uptake, and duration of use in attendees of sexual health services (SHS) in England.

**Methods:**

The Impact Trial was a prospective, open-label, single-arm, multicentre trial conducted at 157 SHS across England between Oct 13, 2017, and July 12, 2020. Clinicians assessed HIV-negative attendees for their risk of HIV acquisition to identify those who were eligible to participate and receive either daily or event-based oral PrEP (tenofovir disoproxil maleate with emtricitabine), as appropriate. Eligible participants were aged 16 years or older, considered HIV-negative on the day of enrolment, and willing to adhere to the trial procedures. Non-trial attendees are mutually exclusive of trial participants and included SHS attendees who were not recruited to the Impact Trial at any point. They include HIV-negative individuals aged 16 years or older who attended a participating SHS at least once after recruitment at that SHS had begun and before Feb 29, 2020. The main outcomes assessed were PrEP need, uptake, and use, and HIV and sexually transmitted infection (STI) incidence. Data are presented up to Feb 29, 2020, before the introduction of COVID-19 control measures. The study is registered with ClinicalTrials.gov, NCT03253757.

**Findings:**

In this analysis, we include 21 356 of 24 268 participants enrolled before Feb 29, 2020. 20 403 participants (95·5%) were men who have sex with men (MSM). Uptake of PrEP among SHS attendees clinically assessed and coded as eligible was 21 292 (57·1%) of 37 289. 18 400 trial participants had at least one post-enrolment visit and a median of 361 days of follow-up (IQR 143−638); 14 039 (75·9%) of these had enough PrEP prescribed to provide protection for 75% of their follow-up time. Among MSM, HIV incidence was 0·13 (95% CI 0·08−0·19) per 100 person-years in trial participants (27 seroconversions) and 0·95 (95% CI 0·88−1·03) per 100 person-years in non-trial attendees (587 seroconversions; proportionate reduction of 86·8%, 95% CI 80·2−91·6). 18 607 bacterial STIs were recorded (incidence 68·1 per 100 person-years in trial participants who were MSM). 4343 (24·4%) MSM participants were diagnosed with two or more STIs, accounting for 14 800 (79·5%) of all 18 607 diagnoses.

**Interpretation:**

PrEP need was higher than initially estimated by an expert stakeholder group. The high proportion of follow-up time protected by PrEP suggests that the need for protection persisted throughout trial participation for most participants. HIV incidence among MSM trial participants was low. The large unmet need for PrEP suggests that greater provision is required to maximise the potential of a national programme. The high incidence of bacterial STIs among participants, concentrated within a subgroup of PrEP users, presents an opportunity for tailored STI control measures.

**Funding:**

NHS England.

## Introduction

In England, there has been a continued decline in the annual number of new HIV diagnoses since 2014, from 5780 then to 2023 in 2021.^1^ Although diagnoses among gay, bisexual, and other men who have sex with men (MSM) represent the highest proportion of new HIV diagnoses (36% in 2021), this population group has also experienced the greatest decline (−77%), from 3200 in 2014 to 721 in 2021.^1^ CD4 cell count back-calculation modelling suggests that the decrease in new diagnoses among MSM is reflective of a true fall in incident infections as a result of combination HIV prevention.^2,3^

Oral tenofovir with emtricitabine as HIV pre-exposure prophylaxis (PrEP) can be highly effective in preventing HIV acquisition.^4–7^ Although data from the PROUD trial reinforced the evidence for efficacy,^8^ information about the level and duration of PrEP need, uptake, and use were required to enable routine commissioning of PrEP in England. On Dec 4, 2016, Public Health England (PHE) and NHS England announced the PrEP Impact Trial, an implementation study to inform the costs of delivering PrEP within specialist sexual health services (SHS).^9^ The primary objectives of the trial were to establish population need, duration of need, PrEP uptake, and duration of use in SHS attendees in England.

## Methods

### Study design

The Impact Trial was a prospective, open-label, single-arm, multicentre trial conducted at 157 of 227 SHS across England between Oct 13, 2017 and July 12, 2020. SHS are dedicated clinics that are free, confidential, and open to anyone without the need to be resident in the local area or registered with (or referred by) a primary health-care physician. All SHS were eligible to join the trial. Participating clinics represented more than 81% of SHS activity in 2019. The Impact Trial was approved by the London Hampstead NHS Research Ethics Committee (reference 17/LO/1134). The protocol is available online. All participants provided written informed consent.

### Participants

Participants were SHS attendees who belonged to one of three population groups: HIV-negative men (cisgender and transgender) and transgender women reporting condomless anal intercourse with men in the previous 3 months and likely to repeat this activity in the subsequent 3 months; HIV-negative partners reporting condomless sex with an HIV-positive person who was not known to be on antiretroviral therapy (ART) and who was not adequately virally suppressed (<200 copies per mL for 6 months or more); and HIV-negative people considered to be at equivalent HIV risk to those in the second group.

Eligible participants were aged 16 years or older, considered HIV-negative on the day of enrolment, and willing to adhere to the trial procedures. Those reporting symptoms consistent with HIV seroconversion or with contraindications to the study drug were not eligible.

Impact Trial participants included in this analysis were SHS attendees who enrolled onto the trial by Feb 29, 2020. Those who tested positive for HIV at or within 6 weeks of enrolment (ie, prevalent HIV infection at baseline) or who had no record of being prescribed PrEP were excluded from statistical analyses.

Non-trial attendees are mutually exclusive of trial participants and included SHS attendees who were not recruited to the Impact Trial at any point. They include HIV-negative individuals aged 16 years or older who attended a participating SHS at least once after recruitment at that SHS had begun and before Feb 29, 2020. HIV-negative individuals were defined as those who had no clinical record indicating a previous HIV diagnosis at the same SHS and who had an HIV test recorded at their first visit after recruitment to the trial had begun, or in the past year if no test was recorded at their first visit. Non-trial attendees who reported obtaining PrEP through another source (eg, self-sourced via the internet) at any point were excluded. Data for non-trial attendees were obtained from the Genitourinary Medicine Clinic Activity Dataset (GUMCAD) only.

In its role in providing infectious disease surveillance, the UK Health Security Agency (formerly Public Health England) has permission to handle data obtained through the GUMCAD STI Surveillance System under Regulation 3 of the Health Service (Control of Patient Information) Regulations 2002; thus specific consent was not required from the non-trial attendees whose data were used in this analysis. Trial participants provided written consent for data linkage.

### Procedures

We collected data using (1) an electronic case report form capturing date of birth, Soundex (coded identifier based on name),^10^ Impact Trial identification number, clinic patient number, self-reported gender at enrolment and gender assigned at birth, number of tablets prescribed, discontinuation status, and serious suspected adverse drug reactions that merited onward reporting to authorities; and (2) the national GUMCAD STI Surveillance System,^11^ capturing age, ethnicity, country of birth, area of residence (categorised into Index of Multiple Deprivation [IMD] quintiles to provide a relative measure of deprivation based on small geographical place of residence), PrEP eligibility, offer, and uptake, prescriptions, and HIV and STI testing and diagnoses. Specific information collected during the clinical consultation (eg, PrEP obtained from another source) is coded and reported according to UK Health Security Agency guidance.^12^ A clinician collected details of the circumstances leading to any incident HIV infections that occurred. GUMCAD is a de-identified dataset that uses a unique, clinic-specific identification code. Participants provided informed consent to link their GUMCAD data to the electronic case report form (appendix pp 2−3).

We tested for STIs, HIV, hepatitis B and C viruses, and serum creatinine (to calculate estimated glomerular filtration rate) at enrolment and every 3 months subsequently, according to national guidelines.^13^ We did urinalysis every 3 months, according to the trial protocol. Investigators provided participants with co-formulated tenofovir disoproxil maleate and emtricitabine (Mylan; Canonsburg, PA, USA) as either daily or event-based dosing.^10^ The choice of regimen dosing was decided following a clinical risk assessment and discussion between the prescribing clinician and participant. Heterosexual men, heterosexual women, and transgender men were offered daily PrEP only. PrEP need was reassessed at each visit and a new prescription dispensed, if required. Participants were offered the opportunity to switch dosing regimen, if appropriate. Those who tested positive for HIV had a confirmatory HIV test, viral load assessment, and resistance tests.

### Outcomes

The main outcome measures were PrEP need, uptake, and use, and incidence of HIV and STIs. Secondary objectives were to establish whether incident HIV infections in PrEP users were due to non-adherence or biological failure of PrEP, to measure change over time in HIV and STI diagnoses and incidence rates among those using PrEP in the trial and other SHS attendees, and to describe PrEP need, uptake, and duration of use (the PrEP prevention care continuum) by different clinic throughput strata and regions in England. Results for the PrEP prevention care continuum by clinic strata will be reported elsewhere. Our analyses include data up to Feb 29, 2020, after which the physical distancing measures introduced for COVID-19 coincided with changes in sexual behaviour, SHS delivery, and PrEP access in England.^14^

### Statistical analyis

A multistakeholder group provided estimates for parameters to inform the sample size, which was initially estimated to be 10 000 participants over 3 years. This number was considered sufficient to estimate the size of the total eligible population and the proportion taking up an offer of PrEP. SHS were allocated a proportion of the trial spaces on the basis of the number of MSM attending during 2016, with a minimum of 20 places allocated to each SHS. Initially, 2000 of the allocated places were reserved for cisgender and transgender women, hetero-sexual cisgender and transgender men, and non-binary people, a group hereafter referred to as women and other populations; this number was reduced to 1000 in response to early rapid uptake of trial places by cisgender MSM and low uptake by women and other populations. Subsequently, there were two increases in the total number of places to 13 000 (July 19, 2018) and 26 000 (Feb 21, 2019) as PrEP need and uptake were greater than originally estimated through expert consensus. Clinics closed or paused enrolment at times when initial recruitment targets were met.

Follow-up time started at the date of enrolment for trial participants and at the first visit after recruitment of participants had started at that site for non-trial attendees. End of follow-up was identified by first date of a positive HIV test. We censored individuals at discontinuation from the trial (participants only) or at the last visit before Feb 29, 2020, if they had no record of a positive HIV test or diagnosis by then.

All time-dependent analyses (ie, duration of HIV risk, duration of PrEP use, and HIV and STI incidence) were restricted to a subset of attendees who had at least one follow-up attendance after enrolment (trial participants) or at least two visits after recruitment at that SHS had started (non-trial attendees), and before Feb 29, 2020.

We estimated overall PrEP need per year within MSM attending SHS by combining the number of all trial participants, non-trial attendees categorised at risk of HIV acquisition, non-trial attendees who were not categorised at risk of HIV acquisition but who sero-converted to HIV during follow-up, and those obtaining PrEP from another source.

PrEP uptake describes the number of attendees clinically assessed as eligible for PrEP who joined the Impact Trial (ie, proportion of SHS attendees ever coded as eligible for PrEP during the analysis period, including at times when enrolment was paused, who began PrEP through participation in the trial). PrEP coverage uses an expanded denominator to include all those categorised as being at risk, whether recognised clinically (and coded) or not. Hence, PrEP uptake is based on GUMCAD eligibility codes and PrEP coverage on the expanded definition of risk described subsequently. PrEP use for all trial participants began from the date of the first prescription and was defined as the proportion of follow-up time with sufficient pills to protect against HIV acquisition. This proportion was calculated as total number of pills prescribed over total follow-up time, regardless of regimen.

We estimated the number of MSM at risk of HIV acquisition during the analysis period using PrEP eligibility, offer, or prescription codes. To account for under-reporting of PrEP eligibility codes, we also used clinical and behavioural markers known to be associated with increased risk of acquiring HIV (markers of higher risk) that were reported during visits in the 12 months before and during the analysis period for HIV incidence.^15,16^ These markers included a rectal bacterial STI diagnosis, use of HIV post-exposure prophylaxis, being a sex worker (ever being a sex worker for the first visit and being a sex worker in the past 12 months for subsequent visits), being a sexual contact of someone diagnosed with HIV or syphilis (attending as a consequence of partner notification), and having had two or more HIV tests in the previous year. We did not estimate the number of women and other populations at risk of HIV acquisition due to the absence of clearly defined markers of higher risk in this diverse population.

Duration of HIV risk was estimated for MSM only and was defined using the interval between two attendances for which, at the most recent attendance, the person was categorised as at risk of HIV acquisition, as described above. If the duration between two attendances was longer than 6 months, a maximum duration of 6 months at risk was considered. For each person, the duration at risk was calculated and expressed as percentage of total follow-up time.

All incidence estimates were restricted to MSM only, due to the low number of HIV and STI infections among women and other populations. HIV incidence rates per 100 person-years (with 95% CI) and Kaplan-Meier curves were generated to compare trial participants and non-trial attendees. Estimates were calculated for all MSM and, as a sensitivity analysis, for MSM categorised as being at risk of HIV acquisition.

We estimated bacterial STI incidence, defined as any diagnosis of chlamydia, gonorrhoea, or early syphilis per 100 person-years, for MSM trial participants and non-trial attendees using date of positive STI test. Concurrent infections of different bacterial STIs were counted separately when estimating the incidence of any bacterial STI. For individual STI incidence estimates, positive results of the same infection at multiple anatomical sites on the same day (eg, pharyngeal and rectal gonorrhoea) were considered as a single infection, whereas concurrent diagnoses of different infections at the same or different anatomical sites were considered separate infections. Tests for different bacterial STIs were counted separately, whereas tests for the same infection at different anatomical sites were counted as a single test. Results of tests for chlamydia and gonorrhoea within 30 days of a previous positive result at the same anatomical site were considered tests of cure and excluded.

To estimate mean STI incidence while accounting for possible differences in testing across groups, we first modelled the observed rates of STI diagnoses and included the number of STI tests recorded during followup as a predictor (empty model).

We compared several distributions for the outcome: Poisson, negative binomial, zero-inflated Poisson, and zero-inflated negative binomial. Zero-inflated negative binomial regression fitted our data best, in terms of Akaike information criterion and Bayesian information criterion.^17^

We then fitted univariate and multivariate zero-inflated negative binomial regression models to estimate the STI incidence rate ratio for MSM trial participants compared with MSM non-trial attendees while adjusting for measured confounders (ie, age group, ethnic group, IMD of residence, region of residence, region of birth, and STI diagnosis in the past year or at first visit). The need for interaction terms between such confounders and inclusion in the trial was tested in the univariate analyses, as was the predictive contribution of each confounder to the inflation factor.

In all regression models, potential differences in service provision were accounted for by estimating a cluster-based variance−covariance matrix to account for within-clinic correlation.

We summarised trends of HIV and STI incidence over follow-up time, both for trial and non-trial participants, for each term (where each term constituted a 90-day period from enrolment or first follow-up). We computed incidence rates having the number of HIV or STI diagnoses as the numerator and the number of people followed up during that term as the denominator. People were assumed to contribute to follow-up time from first to last term appearance.

All data were managed and analysed at the UK Health Security Agency using STATA, version 13 or higher. The study is registered with ClinicalTrials.gov, NCT03253757.

### Role of the funding source

The funder of the trial had no role in trial design, data collection, data analysis, data interpretation, or writing of the manuscript.

## Results

**Figure 1:**
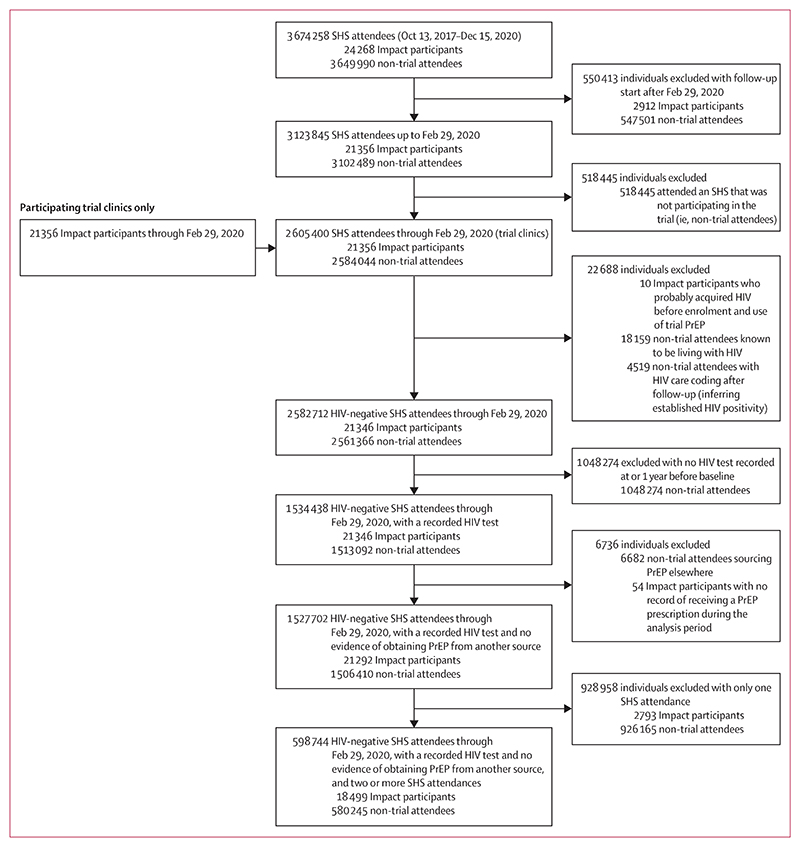
Participant flow chart PrEP=pre-exposure prophylaxis. SHS=sexual health services.

Between Oct 13, 2017, and July 12, 2020, we enrolled 24 268 participants on the Impact Trial, of whom 21 356 were enrolled before Feb 29, 2020, and are included in this analysis (figure 1; appendix p 4). 20 403 trial participants (95·5%) were MSM (table 1). Median age at enrolment was 33 years (IQR 27−42). 16 111 (75·4%) trial participants were of White ethnicity, 13 017 (61·0%) were born in the UK, and 11 300 (52·9%) were living in London. 11 329 (53·0%) were resident in geographical areas belonging to the two most deprived IMD quintiles.

Ten trial participants had a positive HIV test at, or within 6 weeks of, enrolment, indicating probable acquisition of HIV before enrolment and the use of trial PrEP. These individuals were excluded from the statistical analyses. We also excluded 54 trial participants who had no record of receiving a PrEP prescription during the analysis period (figure 1; appendix p 4). Therefore, statistical analyses include 21 292 trial participants with at least one visit and one prescription.

In trial participants, we linked 97·2% of enrolment electronic case report forms to respective GUMCAD records on the attendance day. Discontinuation from the trial during the analysis period was reported for 315 (1·5%) of 21 356 trial participants: 135 (42·9%) withdrew consent, 50 (15·9%) no longer met eligibility criteria, 30 (9·5%) relocated, 27 (8·6%) had HIV seroconversion, 23 (7·3%) withdrew because of adverse events, 21 (6·7%) were withdrawn at clinicians’ discretion, 13 (4·1%) self-reported non-adherence, ten (3·2%) for medical contraindication, five [(1·6%) were lost to followup, and one (0·3%) died (unrelated to PrEP).

1 513 092 other HIV-negative individuals attended participating SHS at least once during the analysis period, of whom 6682 (0·4%) were obtaining PrEP through another source (figure 1; appendix p 4). Those obtaining PrEP through another source were excluded from all statistical analyses (except for estimates of population need), resulting in a comparison group of 1 506 410 non-trial attendees (appendix pp 5−6).

Non-trial attendees were slightly younger, and a lower proportion lived in London, than trial participants. 761 787 (50·6%) were cisgender women, and 562 219 (37·3%) were heterosexual men; only 144 921 (9·6%) were MSM (appendix pp 5−6).

Among 20 349 MSM trial participants with at least one visit, we categorised all as being at risk of HIV acquisition: all met PrEP eligibility criteria, had offer or prescription codes, and 17 680 (86·9%) had additional markers of higher risk (appendix p 7). Among 144 921 MSM non-trial attendees with at least one visit, we categorised 73 930 (51·0%) as being at risk of HIV acquisition: 14 531 (10·0%) had PrEP eligibility criteria, offer, or prescription codes, and 68 998 (47·6%) had additional markers.

Overall, uptake of PrEP among those coded as eligible (ie, clinically assessed and coded as eligible who began the Impact Trial) was 21 292 (57·1%) of 37 289. In MSM, uptake was 20 349 (58·3%) of 34 880, and this was lowest among MSM aged 16−19 years (476 [33·4%] of 1426; table 2). Coverage of PrEP among MSM categorised at risk of HIV acquisition (ie, having markers of higher risk and commenced the Impact Trial) was 20 349 (21·6%) of 94 279. Coverage was lowest in MSM aged 16−19 years (476 [11·3%] of 4219). In women and other populations, 939 (44·5%) of 2111 took up PrEP, with considerable variation by demographic characteristics.

Of 21 292 trial participants, 18 499 (86·9%) had at least one visit after enrolment and are included in time-dependent analyses (figure 1; appendix pp 4, 7−10). Among 1 506 410 non-trial attendees, 580 245 (38·5%) had at least two visits and are included in the time-dependent analyses.

Trial participants with at least one post-enrolment visit (n=18 499) had a median of 361 days of follow-up (IQR 143−638); MSM (n=17 770) had a median of 368 days (146−643) and women and other populations (n=728) had a median of 243 days (105−457).

Non-trial attendees with at least two visits (n=580 245) had a median of 169 days of follow-up (IQR 37−378); MSM (n=85 072) had a median of 205 days (56−433) and women and other populations (n=487 077) had a median of 165 days (35−370).

Among 17 770 MSM trial participants with at least one visit after enrolment, 17 025 (95·8%) were in a period of risk for HIV acquisition during part of their follow-up; 5111 (28·8%) were at risk throughout their entire followup period. Overall, the median proportion of follow-up spent at risk was 91·1% (IQR 75·3−100%) for all MSM trial participants, and 92·0% (78·2−100%) for those who were in a period of risk during part of their follow-up (appendix p 11).

Among 85 072 MSM non-trial attendees with two visits, 51 553 (60·6%) were in a period of risk for HIV acquisition during part of their follow-up; only 8391 (9·9%) were at risk of HIV acquisition throughout their entire follow-up period. The median proportion of follow-up spent at risk was 40·4% (IQR 0−79·1%) for all MSM non-trial attendees, and 72·7% (51·0−91·2%) for those who were in a period of risk during part of their follow-up.

Of 18 499 trial participants with at least one visit after enrolment, 16 599 (89·7%) received more than one prescription during follow-up. The median proportion of follow-up time with enough pills to protect against HIV acquisition was 96·8% (IQR 76·3−100%); the proportion was 96·8% (76·7−100%) for MSM and 93·8% (61·2−100%) for women and other populations; appendix p 11). 14 039 (75·9%) of all 18 499 trial participants were prescribed with enough tablets to protect at least 75% of their follow-up time: 13 563 (76·3%) of 17 770 MSM; 475 (65·2%) of 728 women and other populations.

There were 27 seroconversions (26 MSM and one transgender woman) among trial participants during the analysis period. 14 seroconversions were among participants who had stopped taking PrEP (median time since last PrEP was 8 months, IQR 4−10). A further 12 were among participants who reported adherence at a frequency unlikely to provide adequate protection. Two of these 12 participants had segmental hair analysis, which showed suboptimal levels of PrEP metabolites. Only one participant who acquired HIV reported consistent PrEP use likely to prevent HIV infection.

**Figure 2:**
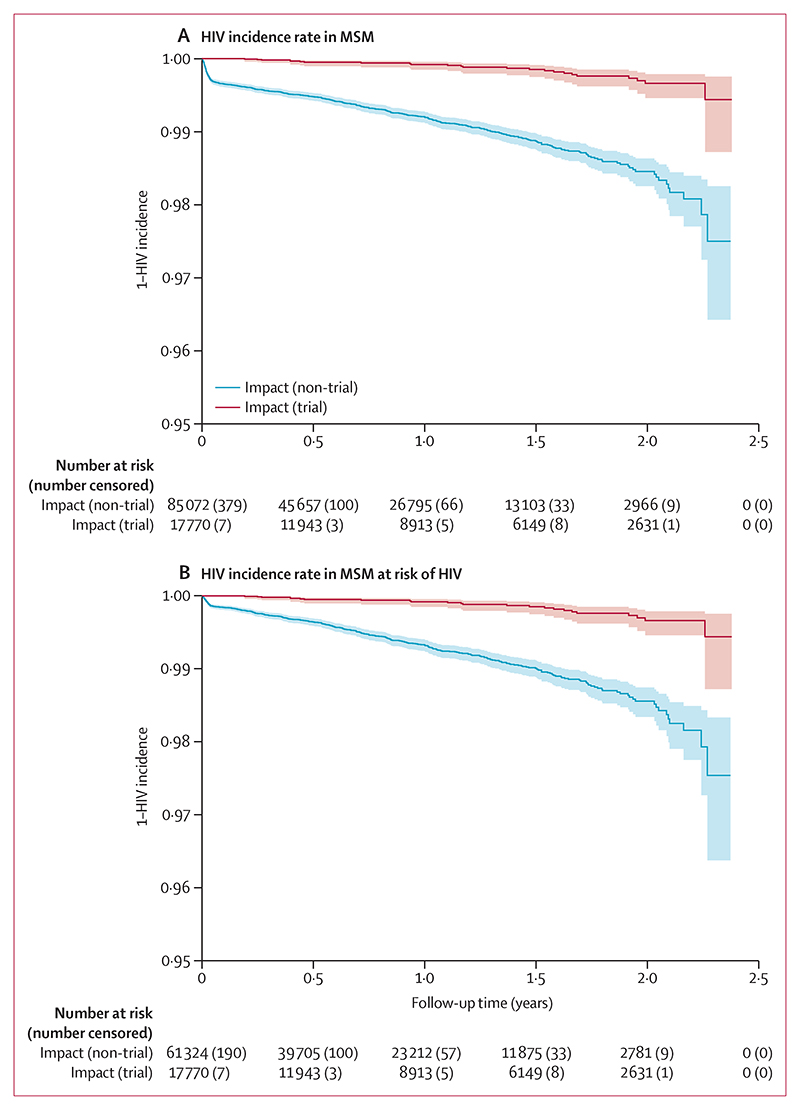
HIV incidence in MSM MSM=men who have sex with men.

Because of the small number of seroconversions in women and other populations (a single participant), we estimated HIV incidence among MSM only. Among the 26 MSM who seroconverted during the analysis period, we included 24 in the incidence analysis; one individual did not have a second visit post-enrolment and the other individual had their follow-up period end at their last visit, which occurred before a later seroconversion. Among 17 770 MSM trial participants, there were 19 075 person-years of follow-up. Overall HIV incidence was 0·13 (95% CI 0·08−0·19) per 100 person-years (figure 2A).

Among 85 072 MSM non-trial attendees, there were 61 605 person-years of follow-up. 587 seroconversions were recorded, giving an HIV incidence estimate of 0·95 (95% CI 0·88−1·03) per 100 person-years. Hence, a proportionate reduction of 86·8% (95% CI 80·2−91·6%) in HIV incidence or, equivalently, a rate difference of 0·83 per 100 person-years (95% CI 0·73−0·92) was observed among MSM trial participants compared with non-trial attendees (figure 2A).

Among 61 324 MSM non-trial attendees categorised as being at risk of HIV acquisition, there were 52 833 person-years of follow-up. 389 seroconversions were recorded, giving an HIV incidence rate of 0·74 per 100 person-years (95% CI 0·67−0·81). A proportionate reduction of 82·9% (95% CI 74·2−89·2%) in HIV incidence or a rate difference of 0·61 per 100 person-years (95% CI 0·52−0·70) was estimated when restricting the analysis to those categorised at risk of HIV (figure 2B).

204 767 STI tests (median 9 per person [IQR 3−18]) were done among MSM trial participants during followup (19 075 person-years). There were 18 607 STI diagnoses (chlamydia, gonorrhoea, and early syphilis), giving a test positivity rate of 9·1%. During follow-up, a mean STI incidence of 97·5 per 100 person-years (95% CI 78·7−121·0) was observed, reducing to 68·1 per 100 person-years (95% CI 50·9−90·9) when accounting for the number of STI tests (table 3). However, more than half of participants (9620 [54·1%] of 17770) had no reported STI diagnoses, whereas 4343 (24·4%) had two or more STI diagnoses accounting for 14 800 (79·5%) of all 18 607 diagnoses (appendix p 13). After adjusting for the number of STI tests, bacterial STI incidence in MSM trial participants was lowest in individuals aged 40 years or older, and highest in those born in Europe; those of Black African, Black Caribbean, or mixed ethnicity; those resident in London; those living in more deprived areas; those who had a bacterial STI diagnosis in the year before enrolment; and those who began a daily PrEP regimen at enrolment (table 4).

Among MSM non-trial attendees, 320 120 STI tests (median 3 per person [IQR 0−6]) and 24 467 diagnoses (7·6% positivity rate) were reported during follow-up. 68 062 (80·0%) had no STI diagnoses reported during follow-up, and 4732 (5·6%) had two or more STI diagnoses. After adjusting for the number of STI tests, overall STI incidence was 24·8 per 100 person-years (95% CI 22·0−27·9; table 3). Although much lower than in trial participants, incidence tended to vary across subgroups in the same way (lower in those aged 40 years or older; higher in those born in Europe, those of Black African or mixed ethnicity, residents of London, and those living in more deprived areas; table 4).

Univariate regression models (results not shown) identified deprivation strata, age, ethnicity, region of residence, country of birth, and a previous STI diagnosis in the year before enrolment as significant confounders. Interaction terms between trial participation and a previous STI diagnosis before enrolment, as well as with region of residence, IMD, and region of birth, were also significant, identifying a smaller detrimental effect of PrEP among attendees without STI diagnosis at enrolment, and those living in the north, south, or abroad (compared with London), and an increased detrimental effect of PrEP in those born in Europe. Finally, not being diagnosed with an STI in the year before enrolment and region of residence significantly explained the probability of having no STIs during follow-up. In the multivariate regression model, the risk of acquiring an STI was 1·66 (95% CI 1·57−1·76) times higher in trial participants, all other factors being equal, than in all non-trial attendees (table 5). A similar association was observed when the comparison group was restricted to non-trial attendees categorised as being at risk of HIV acquisition.

We estimated that the overall MSM SHS attendee need for PrEP was 100 800 (or 58·7% of all HIV-negative MSM attendees at participating clinics, including those sourcing PrEP elsewhere) during the analysis period (42 367 MSM attendees per year); this includes 20 349 trial participants, 73 541 non-trial attendees categorised at risk of HIV acquisition, 587 non-trial attendees who seroconverted (198 who were not categorised as being at risk of HIV), and 6323 attendees sourcing PrEP elsewhere (appendix p 12). The latter represents 94·6% (6323 of 6682) of all individuals who reported obtaining PrEP through another source.

## Discussion

We found that both PrEP need and duration of use markedly exceeded pre-trial estimates, with PrEP uptake by MSM varying widely between key populations and demographics, including age and ethnicity. The high median proportion of follow-up time protected by PrEP suggests that the need for protection persisted throughout trial participation.

There are many reasons why PrEP uptake was low. In particular, enrolment was paused for periods as initial recruitment targets were met, when eligible patients might have attended but were not able to start PrEP. Therefore, this uptake estimate might be lower than expected when PrEP availability is routine. In addition, the absence of PrEP eligibility codes among MSM attendees who had additional markers of higher risk suggests an underuse of the eligibility codes. The lower proportion of younger MSM attendees taking up the offer of PrEP is of particular concern. Coverage was relatively low in MSM. Reasons are likely to be multiple and might reflect a combination of level of awareness of PrEP, insensitive assessments of HIV risk, and concerns related to stigma among both potential participants and clinical staff.^18,19^

Incident HIV infections were rare among MSM trial participants and almost all occurred in the context of having discontinued PrEP. In a single participant, it was not possible to rule out biological failure of PrEP to protect against the acquisition of HIV. HIV incidence was significantly lower among trial participants than among non-trial attendees. A third of HIV infections in MSM non-trial attendees occurred among individuals who were not categorised as being at risk of HIV acquisition at any time during follow-up or the 12 months preceding inclusion. The scarcity of key markers of higher risk reported in current surveillance data highlights the challenges for clinicians to elicit and record relevant behavioural information in MSM attendees.

The many HIV seroconversions in MSM non-trial attendees illustrated the large unmet need for PrEP, both in those categorised at risk of HIV acquisition and those not. Within HIV prevention activities for MSM, a substantial expansion of PrEP access, beyond the level provided during the trial period, will be required to maximise the effectiveness of a national PrEP programme.

As STI diagnoses were not equally distributed among participants, with around half the MSM taking PrEP never being diagnosed with an STI during follow-up and a quarter accounting for 80% of all STIs diagnosed, consideration should be given to adapting screening recommendations and targeting health promotion for those at increased risk.

We delivered the Impact Trial in SHS across all regions of England using the existing national surveillance system as the basic data source. This approach minimised the impact on participating sites and facilitated the collection of large-scale comparable data for trial participants and non-trial attendees. However, the quality of coding varied between clinics, particularly for new PrEP eligibility codes, so these could not be used as the sole data for inferring PrEP need; furthermore, GUMCAD did not capture risk behaviours. Therefore, for MSM, markers of higher risk that could be extracted from an individual’s historical clinical data were used to supplement the new PrEP eligibility codes to identify those at risk. Although these additional markers of higher risk usefully identified the majority of MSM in need of PrEP, HIV seroconversions were observed in a substantial number of those who were not categorised at risk of HIV acquisition. Although some of this insensitivity in risk categorisation might be explained by missing data (individuals moving between clinics, underuse of PrEP eligibility codes, scarcity of GUMCAD behavioural codes), the trial has provided powerful evidence that previous clinical history is an imperfect predictor of future HIV risk when HIV incidence is fairly low.

A principle of SHS in England is that they are open-access, meaning that individuals can self-refer to a clinic of their preference without the need to be a resident of that clinic area. As an additional safeguard for confidentiality of sensitive sexual health data, universal patient identifiers are not used and thus tracking of SHS attendees between clinics is not possible. Therefore, markers of higher risk for individuals who have attended multiple clinics within a year or two will be incomplete, and attendance at different SHS will lead to some over-counting. To mitigate this effect, especially on understanding the duration of PrEP use by those in the trial, a temporary process was created to link a participant’s data if they transferred their PrEP care or temporarily attended another service. The inability to be able to track routine attendees across services might have resulted in an underestimate of HIV and STI incidence in this group.

The surveillance and monitoring system used for data collection does not currently include sexual behavioural data and will not fully capture an individual’s risk. Therefore, it is likely that our calculations underestimate the true need for PrEP among this clinic population in England and further work is required to refine markers of higher risk. An additional cause of underestimation is the large number of attendees who had to be excluded because they did not have a second visit during the analysis period. A further limitation is that we are not able to report bacterial STI incidence by anatomical site systematically across all clinics, restricting to a degree our ability to comment on risk behaviours that might be inferred by such data. Although we provide a measure of STI incidence adjusted by testing patterns comparable between the two groups, we are aware that after the trial, as an increased number of people commence PrEP, prescriptions for 6 months’ worth of tablets might become more routine. As such, patterns of attendance and testing might change, resulting in less frequent attendances and testing, and diminished STI detection rate.

Similar to other studies and demonstration projects,^20,21^ with the exception of a number of studies specifically targeting them, we struggled to identify and recruit cisgender women and heterosexual cisgender men at higher HIV risk. Individuals within these population groups attending SHS in England are generally at much lower risk of HIV acquisition than MSM. A similar number of transgender women were recruited to those in other PrEP studies.^22^ This result is likely to be due to the efforts of various trans-specific services creating a welcoming, supportive service that actively promoted the trial and the parallel community awareness-raising work that occurred during the trial. It was reassuring that there did not appear to be a barrier to access for participants residing in the lower IMD residence bands. For each subgroup of women and other populations at higher HIV risk, it is likely that several barriers existed, for example lower PrEP awareness and knowledge, risk perception, and stigma. Clinic staff might also have failed to identify those who might have benefited from PrEP due to various factors, including clear and specific guidance on this both in the trial protocol and national guidelines.^10^ This reinforces the importance of utilising other elements of HIV combination prevention in addition to PrEP; for example, aiming for very high HIV testing of heterosexual SHS attendees and combining this with effective partner notification and management and treatment as prevention for those with HIV.

The low HIV incidence among participants is in keeping with findings from other implementation studies, although only the EPIC-NSW trial had an equivalent duration of follow-up, reporting 30 seroconversions among the 9709 participants, resulting in an HIV incidence of 1·61 per 1000 person-years.^23^ Almost all seroconversions within the Impact Trial occurred among those who had stopped taking PrEP, adding further real-life data to support the findings from EPIC-NSW that PrEP is almost 100% efficacious for gay and bisexual men who are adherent. The proportionate reduction in HIV incidence between MSM trial participants and non-trial attendees is almost identical to the estimated PrEP effectiveness seen in PROUD (86%), even though the Impact Trial was conducted in the context of a much lower HIV incidence.^8^

The high incidence of STIs among participants is consistent with the experience in other settings using PrEP in gay and bisexual men, as well as studies in young women in sub-Saharan Africa, and supports recommendations in clinical guidelines for regular STI testing for PrEP users.^24–28^ However, as STI diagnoses were concentrated within a subgroup of PrEP users, it might be that these recommendations can be further tailored to the individual and more work is required to best define those at different levels of STI risk. This mirrors findings from the PrEPX trial, which showed an STI incidence of 91·9 per 100 person-years with 25% of participants accounting for 76% of all STIs.^24^

This trial provides important information for service commissioners, providers, clinicians, and guideline authors. Our estimates of PrEP need have illustrated the limitations of current markers and that previous estimates of PrEP need in MSM were substantially lower than those observed in the trial. The expansion of the national reporting system to capture sexual behaviour and a single unique identifier for sexual health clinic attendees would allow better identification of those in need and patterns of use over time, allowing individuals to be followed up across services.

More than half of new HIV diagnoses in the UK are among women, heterosexual men, and transgender, non-binary, and other gender-diverse people.^1^ Although these groups might not, overall, be at high risk, they are relatively under-represented in the trial. This is likely to be due to a combination of factors. Awareness about sexual health, HIV, and combination prevention, including PrEP, is low among some key population groups and needs addressing with evidenced-based, community-appropriate campaigns. Stigma related to both HIV and PrEP is prevalent in many communities and presents a barrier to access; alternatives to daily dosing for some might mitigate some concerns regarding disclosure. Where and by whom PrEP is provided could be expanded, alongside increased support for community signposting and peer-guiding. Within the clinic setting, the role of the health-care professional in discussing and assessing PrEP need with every attendee will be key in improving awareness, uptake, and coverage within these groups. Services and clinicians should ensure this is a routine part of all consultations, especially as it remains unclear how best to identify those at increased risk in some of these under-represented groups.^29–31^ It will be important not to erect barriers to PrEP access by application of strict eligibility criteria; all those at risk of HIV require timely, seamless access. For those who do not access SHS, alternative locations for either provision or signposting should be explored, particularly for those under-represented groups.

A major concern throughout the introduction and scale-up of PrEP provision has been of a resultant rise in STIs due to hypothesised risk compensation. High rates of bacterial STIs were indeed observed in Impact Trial participants; however, most occurred in a minority of participants and were more likely in those with a previous history of STI. This affords us the opportunity to target HIV and STI prevention efforts at those who are more likely to experience an STI, potentially including other prevention interventions, such as doxycycline post-exposure prophylaxis and vaccines. Furthermore, recommendations regarding frequency of screening could be modified for the individual depending on their level of STI risk, potentially releasing capacity and funding to be more effectively directed to those at most risk.

Our trial provided key information on PrEP need, uptake, and HIV and STI incidence across a large number of local sexual health services to inform the national roll-out of PrEP, with a successful outcome in terms of the resulting financial support package. Urgent work is required to ensure equity of access for all who might benefit. Better identification of those who might benefit from PrEP and STI prevention interventions (including screening frequency), along with frictionless and timely access, will allow further refinement of our national combination prevention service and progress to our target of zero HIV transmissions by 2030.^32^

## Supplementary Material

Supplementary appendix

## Figures and Tables

**Table 1:** Characteristics of Impact Trial participants enrolled between Oct 13, 2017, and Feb 29, 2020, by gender and sexual risk

	All participants (n=21 356)	Cisgender MSM (n=20 403)	Cisgender heterosexual men (n=137)	Cisgender women (n=309)	Transgender women (n=319)	Transgender men (n=141)	Non-binary individuals (n=43)
Mean (SD) age, years	35·3 (10·9)	35·3 (10·9)	41·1 (12·4)	34·8 (10·6)	34·5 (11·7)	31·5 (10·2)	29·2 (9·9)
Median (IQR) age, years	33 (27−42)	33 (27−42)	39 (31−51)	33 (27−42)	31 (26−41)	28 (24−38)	27 (23−32)
Age range, years	16−86	16−86	21−76	17−65	17−68	18−78	19−71
Age group							
16−19 years	502 (2·4%)	476 (2·3%)	0	10 (3·2%)	12 (3·8%)	2 (1·4%)	1 (2·3%)
20−24 years	2618 (12·3%)	2462 (12·1%)	7 (5·1%)	44 (14·2%)	51 (16·0%)	38 (27·0%)	15 (34·9%)
25−29 years	4579 (21·4%)	4377 (21·5%)	24 (17·5%)	56 (18·1%)	69 (21·6%)	38 (27·0%)	13 (30·2%)
30−34 years	4066 (19·0%)	3894 (19·1%)	21 (15·3%)	71 (23·0%)	57 (17·9%)	18 (12·8%)	5 (11·6%)
35−39 years	3133 (14·7%)	3023 (14·8%)	20 (14·6%)	30 (9·7%)	43 (13·5%)	14 (9·9%)	3 (7·0%)
40−44 years	2113 (9·9%)	2016 (9·9%)	14 (10·2%)	34 (11·0%)	33 (10·3%)	13 (9·2%)	3 (7·0%)
45−49 years	1786 (8·4%)	1717 (8·4%)	13 (9·5%)	28 (9·1%)	15 (4·7%)	12 (8·5%)	1 (2·3%)
50−54 years	1255 (5·9%)	1203 (5·9%)	11 (8·0%)	22 (7·1%)	14 (4·4%)	4 (2·8%)	1 (2·3%)
55−59 years	696 (3·3%)	663 (3·3%)	16 (11·7%)	9 (2·9%)	8 (2·5%)	0	0
≥60 years	608 (2·9%)	572 (2·8%)	11 (8·0%)	5 (1·6%)	17 (5·3%)	2 (1·4%)	1 (2·3%)
Ethnic group							
White	16 111 (75·4%)	15 547 (76·2%)	67 (48·9%)	182 (58·9%)	190 (59·6%)	99 (70·2%)	25 (58·1%)
Black African	377 (1·8%)	313 (1·5%)	26 (19·0%)	33 (10·7%)	3 (0·9%)	1 (0·7%)	1 (2·3%)
Black Caribbean	351 (1·6%)	331 (1·6%)	9 (6·6%)	8 (2·6%)	1 (0·3%)	2 (1·4%)	0
Black other	134 (0·6%)	125 (0·6%)	1 (0·7%)	4 (1·3%)	2 (0·6%)	2 (1·4%)	0
Asian or Asian British	1092 (5·1%)	1020 (5·0%)	10 (7·3%)	12 (3·9%)	34 (10·7%)	10 (7·1%)	5 (11·6%)
Mixed	926 (4·3%)	854 (4·2%)	6 (4·4%)	21 (6·8%)	30 (9·4%)	10 (7·1%)	5 (11·6%)
Other ethnic group	818 (3·8%)	770 (3·8%)	3 (2·2%)	13 (4·2%)	23 (7·2%)	7 (5·0%)	2 (4·7%)
Unknown	1547 (7·2%)	1443 (7·1%)	15 (11·0%)	36 (11·7%)	36 (11·3%)	10 (7·1%)	5 (11·6%)
Region of birth							
UK	13 017 (61·0%)	12 509 (61·3%)	72 (52·6%)	165 (53·4%)	151 (47·3%)	92 (65·3%)	27 (62·8%)
Europe (excluding UK)	3219 (15·1%)	3126 (15·3%)	5 (3·7%)	39 (12·6%)	26 (8·2%)	15 (10·6%)	8 (18·6%)
Caribbean	99 (0·5%)	96 (0·5%)	2 (1·5%)	0	1 (0·3%)	0	0
Sub-Saharan Africa	587 (2·8%)	521 (2·6%)	28 (20·4%)	33 (10·7%)	3 (0·9%)	1 (0·7%)	1 (2·3%)
South Asia	277 (1·3%)	265 (1·3%)	3 (2·2%)	3 (1·0%)	3 (0·9%)	3 (2·1%)	0
Central America	55 (0·3%)	51 (0·3%)	0	0	4 (1·3%)	0	0
North America	370 (1·7%)	358 (1·8%)	1 (0·7%)	1 (0·3%)	2 (0·6%)	8 (5·7%)	0
South America	741 (3·5%)	656 (3·2%)	1 (0·7%)	22 (7·1%)	55 (17·2%)	6 (4·3%)	1 (2·3%)
Other	1340 (6·3%)	1277 (6·3%)	4 (2·9%)	10 (3·2%)	39 (12·2%)	7 (5·0%)	2 (4·7%)
Unknown	1651 (7·7%)	1544 (7·6%)	21 (15·3%)	36 (11·7%)	35 (11·0%)	9 (6·4%)	4 (9·3%)
Region of residence*							
London	11 300 (52·9%)	10 827 (53·1%)	49 (35·8%)	142 (46·0%)	189 (59·3%)	74 (52·5%)	18 (41·9%)
Midlands and East	2632 (12·3%)	2497 (12·2%)	21 (15·3%)	56 (18·1%)	38 (11·9%)	14 (9·9%)	5 (11·6%)
North	3378 (15·8%)	3247 (15·9%)	23 (16·8%)	46 (14·9%)	39 (12·2%)	15 (10·6%)	8 (18·6%)
South	3709 (17·4%)	3529 (17·3%)	38 (27·7%)	51 (16·5%)	44 (13·8%)	36 (25·5%)	11 (25·6%)
UK other	81 (0·4%)	76 (0·4%)	1 (0·7%)	2 (0·7%)	2 (0·6%)	0	0
Abroad	15 (0·1%)	15 (0·1%)	0	0	0	0	0
Unknown	241 (1·1%)	212 (1·0%)	5 (3·7%)	12 (3·9%)	7 (2·2%)	2 (1·4%)	1 (2·3%)
Index of Multiple Deprivation†														
1 (most deprived)	4433 (20·8%)	4200 (20·6%)	27 (19·7%)	95 (30·7%)	67 (21·0%)	34 (24·1%)	10 (23·3%)
2	6896 (32·3%)	6630 (32·5%)	39 (28·5%)	79 (25·6%)	90 (28·2%)	41 (29·1%)	17 (39·5%)
3	4591 (21·5%)	4382 (21·5%)	37 (27·0%)	55 (17·8%)	73 (22·9%)	36 (25·5%)	7 (16·3%)
4	3057 (14·3%)	2929 (14·4%)	16 (11·7%)	40 (12·9%)	46 (14·4%)	20 (14·2%)	6 (14·0%)
5 (least deprived)	2044 (9·6%)	1961 (9·6%)	12 (8·8%)	26 (8·4%)	34 (10·7%)	8 (5·7%)	2 (4·7%)
Unknown	335 (1·6%)	301 (1·5%)	6 (4·4%)	14 (4·5%)	9 (2·8%)	2 (1·4%)	1 (2·3%)
Regimen at enrolment‡															
Daily	17 239 (80·7%)	16 452 (80·6%)	103 (75·2%)	275 (89·0%)	254 (79·6%)	116 (82·3%)	37 (86·1%)
Event-based dosing	3233 (15·1%)	3129 (15·3%)	24 (17·5%)	11 (3·6%)	47 (14·7%)	17 (12·1%)	5 (11·6%)
Unknown	884 (4·1%)	822 (4·0%)	10 (7·3%)	23 (7·4%)	18 (5·6%)	8 (5·7%)	1 (2·3%)

* The table presents characteristics at enrolment; the total includes four cisgender men with unknown sexual orientation. Enrollees at participating sexual health services from Oct 13, 2017, to Feb 29, 2020 (includes 54 trial participants with no record of receiving a PrEP prescription during the analysis period and ten trial participants who probably acquired HIV before enrolment and use of trial PrEP). LSOA=lower-layer super output area. MSM=men who have sex with men. PrEP=pre-exposure prophylaxis. Region of residence based on LSOA.

† Index of Multiple Deprivation quintiles are based on residence LSOA.

‡ Regimen is based on the coding reported on the enrolment date or, where not reported on enrolment date, the coding reported in the first subsequent visit where reported.

**Table 2:** PrEP uptake and coverage at participating sexual health services (SHS)

	All attendees					Women and other populations*					Men who have sex with men					
	SHS attendees	Ever eligible	Enrolled	Uptake		SHS attendees	Ever eligible	Enrolled	Uptake		SHS attendees	Ever at risk of HIV acquisition	Ever eligible	Enrolled	Uptake	Coverage
Non-trial attendees, n	1506 410	15 997	NA	NA		1 324 261	1 172	NA	NA		144 921	73 930	14 531	NA	NA	NA
Total, n	1 527 702	37 289	21 292	57·1%		1 325 200	2111	939	44·5%		165 270	94 279	34 880	20 349	58·3%	21·6%
Mean (SD) age, years	30·3 (10·5)	34·3 (10·3)	35·3 (10·3)	··		29·3 (10·2)	34·3 (12·4)	34·9 (11·4)	··		33·7 (11·9)	33·6 (11·5)	34·3 (11·4)	35·3 (10·9)	··	··
Median (IQR) age, years	28 (22−35)	32 (26−41)	33 (27−42)	··		27 (22−35)	31 (25−41)	32 (26−42)	··		31 (25−40)	31 (25−40)	32 (26−41)	33 (27−42)	··	··
Age range, years	16−99	16−91	16−86	··		16−99	16−91	17−76	··		16−94	16−89	16−89	16−86	··	··
Age group																
16−19 years	146 833	1536	501	32·6%		135 003	97	24	24·7%		8364	4216	1423	476	33·5%	11·3%
20−24 years	394 698	5932	2603	43·9%		354 292	386	152	39·4%		30 898	16 805	5475	2450	44·7%	14·6%
25−29 years	346 069	8069	4558	56·5%		301 279	442	197	44·6%		36 276	21 376	7553	4359	57·7%	20·4%
30−34 years	232 080	6596	4059	61·5%		198 620	343	172	50·1%		27 829	16 528	6223	3887	62·5%	23·5%
35−39 years	149 678	4878	3128	64·1%		126 541	235	110	46·8%		19 481	11 697	4617	3018	65·4%	25·8%
40−44 years	91 130	3225	2109	65·4%		76 430	198	97	49·0%		12 470	7393	3000	2012	67·1%	27·2%
45−49 years	65 966	2649	1784	67·3%		54 207	127	67	52·8%		10 214	5817	2498	1717	68·7%	29·5%
50−54 years	45 941	1968	1250	63·5%		36 870	116	52	44·8%		7934	4378	1845	1198	64·9%	27·4%
55−59 years	27 660	1171	694	59·3%		21 736	79	33	41·8%		5211	2756	1083	661	61·0%	24·0%
≥ 60 years	27 474	1265	606	47·9%		20 093	88	35	39·8%		6589	3313	1163	571	49·1%	17·2%
Unknown	173	0	0	NA		129	0	0	NA		4	0	0	0	NA	NA
Ethnic group																
White	1 063 999	28 157	16 061	57·0%		920 773	1381	555	40·2%		124 041	70 996	26 574	15 505	58·3%	21·8%
Black African	72 432	694	376	54·2%		68 316	146	64	43·8%		2465	1548	542	312	57·6%	20·2%
Black Caribbean	54 560	672	350	52·1%		50 499	48	19	39·6%		2591	1698	623	331	53·1%	19·5%
Black other	16 458	226	134	59·3%		15 199	24	9	37·5%		858	544	199	125	62·8%	23·0%
Asian or Asian British	80 332	2039	1092	53·6%		69 726	127	71	55·9%		8817	5355	1899	1020	53·7%	19·0%
Mixed	72 650	1601	923	57·7%		64 274	133	72	54·1%		6816	4031	1454	851	58·5%	21·1%
Other ethnic group	40 658	1401	817	58·3%		33 274	100	48	48·0%		6345	3809	1288	769	59·7%	20·2%
Unknown	126 613	2499	1539	61·6%		103 139	152	101	66·4%		13 337	6298	2301	1436	62·4%	22·8%
Region of birth																
UK	1 029 296	23 598	12 978	55·0%		910 639	1253	500	39·9%		103 300	58 430	22 181	12 477	56·3%	21·4%
Europe (excluding UK)	147 492	5208	3212	61·7%		122 743	230	93	40·4%		22 374	14 142	4939	3119	63·2%	22·1%
Caribbean	13 047	176	98	55·7%		11 958	16	2	12·5%		707	436	160	96	60·0%	22·0%
Sub-Saharan Africa	60 351	968	585	60·4%		55 782	144	66	45·8%		3257	2054	816	519	63·6%	25·3%
South Asia	22 450	501	276	55·1%		19 581	22	11	50·0%		2437	1442	476	265	55·7%	18·4%
Central America	1 460	84	54	64·3%		1021	8	4	50·0%		408	256	76	50	65·8%	19·5%
North America	11 103	535	368	68·8%		8644	26	12	46·2%		2238	1273	497	356	71·6%	28·0%
South America	15 838	1129	741	65·6%		11 757	130	85	65·4%		3702	2483	995	656	65·9%	26·4%
Other	55 335	2172	1337	61·6%		45 108	129	62	48·1%		9133	5623	2023	1274	63·0%	22·7%
Unknown	171 330	2918	1643	56·3%		137 967	153	104	68·0%		17 714	8140	2717	1537	56·6%	18·9%
Region of residence†																
London	439 910	16 268	11 275	69·3%		359 830	855	469	54·9%		68 143	42 082	15 304	10 805	70·6%	25·7%
Midlands and East	388 392	5650	2615	46·3%		346 819	316	131	41·5%		31 257	15 787	5297	2483	46·9%	15·7%
North	324 174	6639	3366	50·7%		284 302	307	129	42·0%		31 419	17 450	6265	3237	51·7%	18·6%
South	340 747	8042	3700	46·0%		305 500	573	178	31·1%		30 122	17 131	7395	3522	47·6%	20·6%
UK other	12 954	204	81	39·7%		11 105	12	5	41·7%		1630	729	190	76	40·0%	10·4%
Abroad	1293	29	15	51·7%		1047	2	0	0·0%		220	94	27	15	55·6%	16·0%
Unknown	20 232	457	240	52·5%		16 597	46	27	58·7%		2479	1006	402	211	52·5%	21·0%
Index of Multiple Deprivation‡																
1 (most deprived)	354 080	7935	4418	55·7%		310 535	501	228	45·5%		34 129	19 744	7360	4190	56·9%	21·2%
2	388 337	11 360	6876	60·5%		329 509	568	266	46·8%		48 103	28 421	10 706	6610	61·7%	23·3%
3	303 485	7835	4576	58·4%		261 880	414	204	49·3%		34 374	19 910	7355	4371	59·4%	22·0%
4	244 297	5602	3053	54·5%		214 094	343	127	37·0%		25 516	14 265	5218	2926	56·1%	20·5%
5 (least deprived)	203 037	3869	2035	52·6%		180 442	225	82	36·4%		18 823	10 113	3624	1952	53·9%	19·3%
Unknown	34 466	688	334	48·5%		28 740	60	32	53·3%		4325	1826	617	300	48·6%	16·4%
Gender and sexual orientation§																
Cisgender women	762 092	603	305	50·6%		NA	NA	NA	NA		NA	NA	NA	NA	NA	NA
Cisgender heterosexual men	562 354	942	135	14·3%		NA	NA	NA	NA		NA	NA	NA	NA	NA	NA
Transgender women	462	359	318	88·6%		NA	NA	NA	NA		NA	NA	NA	NA	NA	NA
Transgender men	249	164	138	84·1%		NA	NA	NA	NA		NA	NA	NA	NA	NA	NA
Cisgender men who have sex with men	165 270	34 880	20 349	58·3%		NA	NA	NA	NA		NA	NA	NA	NA	NA	NA

* Data are presented on HIV-negative attendees accessing a participating specialist SHS at least once after recruitment at that SHS had begun and before Feb 29, 2020, with a recorded HIV test and no evidence of obtaining PrEP from another source; these data exclude 54 trial participants with no record of receiving a PrEP prescription during the analysis period and ten trial participants who probably acquired HIV before enrolment and use of trial PrEP. Ever at risk of HIV acquisition refers to individuals with PrEP eligibility, offer or prescription codes, or markers of higher risk from start of follow-up to Feb 29, 2020; ever eligible refers to individuals ever coded as eligible for PrEP; uptake refers to those enrolled or ever eligible, whereas coverage refers to enrolled or ever at risk of HIV acquisition. LSOA=lower-layer super output area. MSM=men who have sex with men. NA=not applicable. PrEP=pre-exposure prophylaxis. Other populations include cisgender women, cisgender heterosexual men, transgender women, transgender men, and non-binary individuals (not specified in non-trial attendees).

† Region of residence is based on LSOA.

‡ Index of Multiple Deprivation quintiles are based on residence LSOA.

§ Data for cisgender men of unknown sexual orientation and non-binary individuals are not presented (but are included in the overall total).

**Table 3:** Bacterial STI incidence per 100 person-years among MSM trial participants and non-trial attendees

	MSM Impact participants (n=17 770)	MSM non-trial attendees (all; n=85 072)	MSM non-trial attendees ever at risk of HIV acquisition (n=61 324)
**Unadjusted**			
Any STI	97·5 (78·7−121·0)	39·7 (33·9−46·5)	42·8 (37·0−49·3)
Chlamydia	42·2 (34·8−51·2)	15·2 (13·1−17·6)	16·6 (14·6−18·8)
Gonorrhoea	48·4 (37·8−62·1)	20·4 (16·6−24·9)	22·1 (18·4−26·5)
Syphilis	6·90 (6·07−7·83)	4·14 (3·72−4·62)	4·08 (3·71−4·50)
**Adjusted for number of STI tests**			
Any STI	68·1 (50·9−90·9)	24·8 (22·0−27·9)	28·3 (26·1−30·7)
Chlamydia	28·4 (20·7−38·9)	6·04 (5·23−6·98)	7·63 (7·05−8·26)
Gonorrhoea	35·0 (24·5−50·0)	8·14 (6·52−10·2)	10·2 (8·90−11·7)
Syphilis	4·47 (3·32−6·01)	1·80 (1·41−2·29)	1·82 (1·39−2·39)

Data are presented with 95% CIs. Incidence estimates are based on HIV-negative attendees accessing a participating sexual health service with at least one follow-up attendance after enrolment (Impact participants) or at least two visits after recruitment at that service had started (non-trial attendees) to Feb 29, 2020, with a recorded HIV test and no evidence of obtaining PrEP from another source; the analysis excludes 54 trial participants with no record of receiving a PrEP prescription during the analysis period, ten trial participants who probably acquired HIV before enrolment and the use of trial PrEP, and 928958 individuals with only one sexual health service attendance (2793 trial participants and 926 165 non-trial attendees). Ever at risk of HIV acquisition refers to individuals with PrEP eligibility, offer or prescription codes, or markers of higher risk from the start of follow-up to Feb 29, 2020. MSM=men who have sex with men. PrEP=pre-exposure prophylaxis. STI=sexually transmitted infection.

**Table 4:** Mean bacterial STI incidence per 100 person-years among MSM trial participants and non-trial attendees by demographic and clinical characteristics

	MSM Impact participants (n=17 770)			MSM non-trial attendees (n=85 072)	
	Incidence per 100 person-years	Incidence rate ratio		Incidence per 100 person-years	Incidence rate ratio
**Unadjusted**					
Age group					
16−24 years	111·7 (93·7−133·2)	1·00 (ref)		44·5 (39·0−50·8)	1·00 (ref)
25−29 years	118·0 (91·0−153·1)	1·06 (0·96−1·17)		44·6 (36·9−53·9)	1·00 (0·93−1·08)
30−34 years	107·1 (87·2−131·5)	0·96 (0·90−1·02)		43·3 (37·3−50·4)	0·97 (0·93−1·02)
35−39 years	96·8 (78·1−120·0)	0·87 (0·81−0·93)		35·9 (29·9−43·1)	0·81 (0·74−0·88)
≥40 years	75·5 (64·2−88·8)	0·68 (0·64−0·72)		30·6 (26·7−35·2)	0·69 (0·65−0·73)
World region of birth					
UK	87·1 (72·3−105·0)	1·00 (ref)		37·3 (32·7−42·5)	1·00 (ref)
Europe (excluding UK)	130·0 (108·5−155·9)	1·49 (1·41−1·58)		47·3 (40·2−55·5)	1·27 (1·19−1·34)
Sub-Saharan Africa	104·1 (81·5−127·8)	1·19 (1·09−1·31)		42·8 (37·1−49·4)	1·15 (1·04−1·27)
Other	101·5 (80·6−127·8)	1·16 (1·08−1·25)		40·9 (33·2−50·5)	1·10 (0·97−1·24)
STI in past year					
No previous STI and no STI diagnosis at enrolment	72·0 (60·7−73·8)	1·00 (ref)		29·4 (24·5−35·3)	1·00 (ref)
Unknown previous STI and no STI diagnosis at enrolment	61·8 (54·4−70·2)	0·86 (0·76−0·96)		34·8 (30·7−39·5)	1·18 (1·08−1·29)
	
Previous STI or STI diagnosis at enrolment, or both	132·4 (110·0−159·3)	1·84 (1·78−1·90)		61·1 (52·5−71·2)	2·08 (1·98−2·18)
Index of Multiple Deprivation (residence)*					
1 (most deprived)	99·7 (82·0−121·4)	1·00 (ref)		42·6 (38·5−47·1)	1·00 (ref)
2	107·6 (85·7−135·2)	1·08 (1·02−1·14)		44·1 (36·9−52·7)	1·04 (0·95−1·14)
3	97·6 (80·1−118·9)	0·98 (0·93−1·02)		38·0 (31·9−45·2)	0·89 (0·81−0·98)
4	86·0 (70·7−104·7)	0·86 (0·82−0·91)		36·1 (30·6−42·6)	0·85 (0·78−0·92)
5 (least deprived)	77·0 (65·7−90·3)	0·77 (0·71−0·84)		31·1 (26·4−36·7)	0·73 (0·66−0·81)
Unknown	54·6 (37·0−80·4)	0·55 (0·41−0·74)		33·7 (28·9−39·4)	0·79 (0·68−0·92)
Region of residence					
London	114·2 (94·7−137·6)	1·00 (ref)		46·4 (38·3−56·1)	1·00 (ref)
Midlands and East	73·4 (62·6−86·0)	0·64 (0·53−0·79)		32·8 (30·3−35·6)	0·71 (0·59−0·85)
North	77·0 (68·6−86·5)	0·67 (0·54−0·84)		38·0 (34·5−41·8)	0·82 (0·66−1·01)
South	71·2 (64·7−78·4)	0·62 (0·52−0·75)		31·8 (28·8−35·1)	0·69 (0·58−0·82)
Other	54·9 (37·6−80·3)	0·48 (0·35−0·66)		33·9 (29·0−39·5)	0·73 (0·59−0·90)
Ethnic group					
White	97·1 (77·5−121·6)	1·00 (ref)		38·6 (32·9−45·4)	1·00 (ref)
Black African	115·7 (97·0−137·9)	1·19 (1·05−1·35)		50·8 (45·5−56·7)	1·31 (1·13−1·53)
Black Caribbean	120·7 (96·8−150·5)	1·24 (1·10−1·41)		49·9 (46·2−53·8)	1·29 (1·11−1·15)
Black other	92·3 (61·8 −137·6)	0·95 (0·78−1·17)		45·5 (37·4−55·2)	1·18 (0·99−1·40)
Asian or Asian British	99·6 (88·8−111·6)	1·03 (0·87−1·21)		37·9 (32·8−43·7)	0·98 (0·91−1·05)
Mixed	121·2 (102·0−144·1)	1·25 (1·10−1·42)		48·3 (39·3−59·3)	1·25 (1·17−1·34)
Other ethnic group	101·4 (81·4−126·4)	1·04 (0·96−1·14)		41·7 (37·8−45·9)	1·08 (0·98−1·18)
Unknown	73·5 (60·4−89·4)	0·76 (0·69−0·83)		39·9 (30·6−52·0)	1·03 (0·90−1·19)
Regimen at enrolment†					
Daily	100·3 (81·0−124·1)	1·00 (ref)			··
Event-based dosing	85·0 (66·1−109·2)	0·85 (0·80−0·89)			··
Unknown	73·3 (54·5−98·5)	0·73 (0·50−1·06)			··
**Adjusted for number of STI tests**					
Age group					
16−24 years	73·8 (56·6−96·2)	1·00 (ref)		28·4 (24·0−33·5)	1·00 (ref)
25−29 years	74·4 (67·0−102·1)	0·98 (0·91−1·06)		26·5 (22·9−30·6)	0·96 (0·91−1·02)
30−34 years	74·2 (52·0−105·9)	0·87 (0·82−0·92)		27·4 (24·3−30·9)	0·92 (0·88−0·96)
35−39 years	67·1 (48·0−93·7)	0·77 (0·72−0·82)		23·3 (20·0−27·0)	0·77 (0·71−0·83)
≥40 years	47·0 (37·7−58·6)	0·60 (0·57−0·64)		18·6 (16·9−20·5)	0·67 (0·64−0·71)
World region of birth					
UK	63·7 (50·3−80·6)	1·00 (ref)		24·1 (22·0−26·4)	1·00 (ref)
Europe (excluding UK)	94·8 (69·0−130·2)	1·40 (1·34−1·46)		27·4 (23·7−31·5)	1·17 (1·10−1·24)
Sub-Saharan Africa	79·6 (55·5−114·0)	1·12 (1·03−1·22)		25·6 (17·8−36·8)	1·08 (0·99−1·19)
Other	65·9 (44·8−97·1)	1·12 (1·04−1·20)		25·9 (20·4−32·8)	1·07 (0·96−1·19)
STI in past year					
No previous STI and no STI diagnosis at enrolment	51·4 (38·5−68·7)	1·00 (ref)		13·1 (10·2−16·8)	1·00 (ref)
Unknown previous STI and no STI diagnosis at enrolment	37·7 (28·8−49·3)	0·89 (0·82−0·98)		23·0 (19·6−26·9)	1·42 (1·33−1·52)
Previous STI or STI diagnosis at enrolment, or both	96·6 (71·3−130·8)	1·76 (1·71−1·81)		38·3 (34·8−42·1)	2·09 (1·97−2·22)
Index of Multiple Deprivation (residence)*					
1 (most deprived)	72·8 (49·8−106·5)	1·00 (ref)		27·0 (23·8−30·7)	1·00 (ref)
2	80·1 (57·5−111·7)	1·05 (0·99−1·12)		27·3 (22·9−32·7)	1·01 (0·93−1·10)
3	72·4 (57·3−91·6)	0·97 (0·93−1·02)		23·6 (21·0−26·6)	0·89 (0·82−0·96)
4	54·8 (42·0−71·5)	0·86 (0·82−0·90)		23·2 (20·4−26·3)	0·85 (0·79−0·92)
5 (least deprived)	61·4 (47·2−79·9)	0·80 (0·74−0·86)		18·8 (16·5−21·3)	0·75 (0·69−0·82)
Unknown	6·90 (3·57−13·3)	0·61 (0·48−0·77)		22·0 (15·8−30·6)	0·86 (0·73−1·00)
Region of residence					
London	89·8 (71·3−113·1)	1·00 (ref)		28·3 (24·3−32·9)	1·00 (ref)
Midlands and East	38·6 (20·8−71·8)	0·72 (0·61−0·86)		18·4 (14·1−23·9)	0·79 (0·67−0·94)
North	59·4 (41·3−85·4)	0·75 (0·61−0·91)		26·2 (21·3−32·3)	0·91 (0·75−1·10)
South	66·5 (54·0−82·0)	0·68 (0·58−0·81)		20·8 (16·5−26·2)	0·77 (0·65−0·90)
Other	6·84 (3·54−13·2)	0·55 (0·43−0·71)		21·8 (15·7−30·4)	0·84 (0·68−1·03)
Ethnic group					
White	66·4 (50·6−87·0)	1·00 (ref)		23·7 (21·2−26·4)	1·00 (ref)
Black African	99·8 (67·3−147·8)	1·16 (1·03−1·30)		36·6 (29·0−46·1)	1·22 (1·05−1·41)
Black Caribbean	107·7 (68·9−168·3)	1·26 (1·11−1·42)		33·6 (25·0−45·2)	1·17 (0·99−1·37)
Black other	71·1 (40·8−123·9)	0·92 (0·78−1·10)		33·8 (22·0−52·0)	1·10 (0·93−1·31)
Asian or Asian British	62·4 (43·4−89·8)	1·05 (0·91−1·21)		26·1 (21·1−32·3)	0·96 (0·91−1·02)
Mixed	101·4 (69·8−147·2)	1·23 (1·09−1·39)		29·7 (25·1−35·3)	1·22 (1·14−1·29)
Other ethnic group	90·7 (67·1−122·5)	1·00 (0·92−1·09)		29·7 (24·2−36·4)	1·04 (0·93−1·15)
Unknown	44·1 (24·5−79·6)	0·79 (0·73−0·86)		25·4 (18·9−34·1)	1·08 (0·94−1·25)
Regimen at enrolment†					
Daily	77·1 (56·1−105·9)	1·00 (ref)			··
Event-based dosing	46·4 (39·3−54·7)	0·91 (0·86−0·97)			··
Unknown	24·1 (14·6−39·8)	0·89 (0·66−1·20)			··

Data are presented with 95% CIs. Incidence estimates are based on HIV-negative attendees accessing a participating sexual health service with at least one follow-up attendance after enrolment (Impact participants) or at least two visits after recruitment at that service had started (non-trial attendees) to Feb 29, 2020, with a recorded HIV test and no evidence of obtaining PrEP from another source; the analysis excludes 54 trial participants with no record of receiving a PrEP prescription during the analysis period, ten trial participants who probably acquired HIV before enrolment and use of trial PrEP, and 928958 individuals with only one sexual health service attendance (2793 Impact participants and 926 165 non-trial attendees). MSM=men who have sex with men. PrEP=pre-exposure prophylaxis. STI=sexually transmitted infection.

* Index of Multiple Deprivation quintiles are based on residence lower-layer super output area.

† The regimen is based on coding reported on enrolment date or, when not reported on enrolment date, the coding reported in the first subsequent visit when reported.

**Table 5:** Multivariate regression model comparing mean STI incidence in MSM trial participants and non-trial attendees, after adjusting for number of tests and all relevant confounders

	Incidence rate ratio (95% CI)	p value
Total STI tests	0·98 (0·96 to 1·00)	0·094
STI tests (squared)	1·0011 (1·0005 to 1·0016)	0·0001
Total STI diagnoses		
MSM non-trial attendee	1 (ref)	··
MSM Impact participant	1·66 (1·57 to 1·76)	<0·0001
Index of Multiple Deprivation (residence)*		
1 (most deprived)	1 (ref)	· ·
2	1·00 (0·96 to 1·05)	0·96
3	0·94 (0·90 to 0·98)	0·0078
4	0·91 (0·86 to 0·96)	0·0007
5 (least deprived)	0·86 (0·79 to 0·94)	0·0006
Unknown	0·60 (0·23 to 1·59)	0·31
Age group		
16−24 years	1 (ref)	· ·
25−29 years	0·96 (0·92 to 1·00)	0·062
30−34 years	0·89 (0·86 to 0·92)	<0·0001
35−39 years	0·78 (0·75 to 0·82)	<0·0001
≥40 years	0·70 (0·67 to 0·73)	<0·0001
Ethnic group		
White	1 (ref)	··
Black African	1·05 (0·94 to 1·17)	0·39
Black Caribbean	1·10 (0·99 to 1·22)	0·073
Black other	0·93 (0·82 to 1·07)	0·34
Asian or Asian British	0·95 (0·88 to 1·02)	0·16
Mixed	1·14 (1·09 to 1·19)	<0·0001
Other ethnic group	0·98 (0·91 to 1·06)	0·61
Unknown	0·93 (0·86 to 1·01)	0·096
STI in past year		
No previous STI and no STI diagnosis at enrolment	1 (ref)	··
Unknown previous STI and no STI diagnosis at enrolment	1·43 (1·34 to 1·53)	<0·0001
Previous STI or STI diagnosis at enrolment, or both	1·71 (1·63 to 1·81)	<0·0001
Region of residence		
London	1 (ref)	··
Midlands and East	0·89 (0·81 to 0·98)	0·016
North	0·88 (0·79 to 0·99)	0·027
South	0·82 (0·74 to 0·90)	<0·0001
Other	1·42 (0·56 to 3·60)	0·46
Region of birth		
UK	1 (ref)	··
Europe (excluding UK)	1·07 (1·02 to 1·14)	0·0088
Sub-Saharan Africa	1·07 (0·97 to 1·17)	0·19
Other	1·01 (0·93 to 1·09)	0·88
Interaction terms		
Interaction between Impact participant and STI in past year		
Impact participant and no previous STI, no STI diagnosis at enrolment	1 (ref)	· ·
Impact participant and unknown previous STI, no STI diagnosis at enrolment	0·63 (0·56 to 0·71)	<0·0001
Impact participant and previous STI or STI diagnosis at enrolment, or both	0·95 (0·90 to 1·01)	0·11
Interaction between Impact and region of birth		
Impact participant and UK	1 (ref)	· ·
Impact participant and Europe	1·16 (1·09 to 1·22)	<0·0001
Impact participant and sub-Saharan Africa	1·00 (0·90 to 1·10)	0·98
Impact participant and other	1·07 (0·99 to 1·16)	0·11	
Constant (y-intercept)	54·2 (50·5 to 58·1)	<0·0001
ln(time enrolled)	1·00	· ·
Inflation factor		
Total STI tests	−0·60 (−0·88 to −0·32)	<0·0001
STI tests (squared)	0·0106 (0·0057 to 0·0154)	<0·0001
Time enrolled	190·1 (158·0 to 222·2)	<0·0001
STI in past year		
Unknown previous STI, no STI diagnosis at enrolment	0·21 (−0·04 to 0·47)	0·10
Previous STI or STI diagnosis at enrolment	−0·86 (−1·09 to −0·64)	<0·0001
Interaction between Impact and STI in past year		
Impact participant and no previous STI, no STI diagnosis at enrolment	0·45 (−0·14 to 1·05)	0·14
Impact participant and unknown previous STI, no STI diagnosis at enrolment	−0·25 (−0·69 to 0·20)	0·28
Impact participant and previous STI or STI diagnosis at enrolment	0·68 (0·33 to 1·03)	0·0001
Region of residence		
Midlands and East	0·46 (0·36 to 0·87)	0·033
North	0·17 (−0·20 to 0·54)	0·38
South	0·22 (−0·17 to 0·61)	0·26
Other	0·42 (−0·26 to 1·11)	0·23
Constant (y·intercept)	−0.60 (−1·46 to 0·26)	0·17

Incidence estimates are based on HIV-negative attendees accessing a participating sexual health service with at least one follow-up attendance after enrolment (Impact participants) or at least two visits after recruitment at that service had started (non-trial attendees) to Feb 29, 2020, with a recorded HIV test and no evidence of obtaining PrEP from another source; the analysis excludes 54 trial participants with no record of receiving a PrEP prescription during the analysis period, ten trial participants who probably acquired HIV before enrolment and the use of trial PrEP, and 928958 individuals with only one sexual health service attendance (2793 Impact participants and 926 165 non-trial attendees). MSM=men who have sex with men. PrEP=pre-exposure prophylaxis. STI=sexually transmitted infection.

* Index of Multiple Deprivation quintiles are based on residence lower-layer super output area.

## Data Availability

Data collected for the study will be made available to other researchers following approval of their proposal by the Trial Steering Committee and UK Health Security Agency Impact Trial Data Sharing Review Panel in accordance with the HIV PrEP Impact Trial: PHE Data Handling Plan (February, 2020) and a signed data sharing agreement. Data will include de-identified participant data and a data dictionary. These data will be made available once the main trial manuscripts have been published for a period of 2 years. The study protocol and statistical analysis plan are also available upon request. Applications should be made via email to ann.sullivan2@nhs.net.

## Research in context

### Evidence before this study

Oral tenofovir disoproxil maleate with emtricitabine as HIV pre-exposure prophylaxis (PrEP) is highly effective at preventing HIV acquisition in all key population groups when taken as directed and recommended by WHO as part of combination HIV prevention. In England, the PROUD study enrolled 544 participants between November, 2012, and April, 2014, to test the feasibility of a large-scale trial. Because of the unexpectedly high incidence of HIV, the study was able to show effectiveness of PrEP and stopped early. However, national and local government required further information to commission wider-scale roll-out, including the estimated level of PrEP need, uptake, and use within PrEP programmes. We searched PubMed on July 28, 2022, with no date or language restrictions, using the terms ([“pre-exposure prophylaxis” OR “PrEP” OR “preexposure prophylaxis”] AND [“HIV”]), limited to observational studies. This search identified 27 publications. Although some reported proportions of their populations meeting PrEP eligibility and duration of PrEP use during the period of observation, none of these were within England-based populations or at numbers sufficiently large to provide robust estimates required for commissioning in England.

### Added value of this study

The Impact Trial is one of the largest implementation studies of PrEP and enrolled more than 24 000 participants over 3 years. It provides important estimates of PrEP eligibility, uptake, and use among key population groups in England, as well as more refined estimates of sexually transmitted infection (STI) and HIV incidence among PrEP users. The trial showed a large unmet need for PrEP in England, including among cisgender gay, bisexual, and other men who have sex with men. Among those taking up an offer of PrEP, the need for protection persisted throughout the trial period. The data support earlier evidence that PrEP is highly effective; incident HIV infections were rare and almost all occurred in the context of having discontinued PrEP. Although STI incidence was high, this was concentrated among a minority of participants and was more likely among those with a history of STIs than among those without.

### Implications of all the available evidence

Our findings have been used to inform routine commissioning and delivery of PrEP in England since October, 2020. This change is an important step towards the elimination of HIV transmission as part of combination HIV prevention. We also confirmed that PrEP is highly effective in a real-world setting across several years of use. However, further work is required to optimise PrEP delivery to all who could benefit, particularly those with recognised behavioural and clinical factors associated with an increased risk of HIV.
