# Supplementary material for: HIV pre-exposure prophylaxis and its implementation in the PrEP Impact Trial in England: a pragmatic health technology assessment
Source: Lancet HIV. Author manuscript; Available in PMC 2024 Dec 2. (PMC7616873; doi:10.1016/S2352-3018(23)00256-4)
Supplement: Supplementary appendix [file EMS200044-supplement-Supplementary_appendix.pdf]

THE LANCET
HIV

Supplementary appendix

This appendix formed part of the original submission and has been peer reviewed. We post it as supplied by the authors.

Supplement to: Sullivan AK, Saunders J, Desai M, et al. HIV pre-exposure prophylaxis and its implementation in the PrEP Impact Trial in England: a pragmatic health technology assessment. *Lancet HIV* 2023; **10:** e790–806.

A pragmatic health technology assessment of HIV Pre exposure prophylaxis and implementation: Findings from the PrEP Impact Trial in England

Appendix

- **Data linkage methods (page 2)**
- **Appendix Figure 1 (page 2)**
- **Appendix Table 1 (page 3)**
- **Appendix Table 2 (page 4)**
- **Appendix Table 3 (pages 5 to 6)**
- **Appendix Table 4 (page 7)**
- **Appendix Table 5 (pages 8 to 10)**
- **Appendix Figure 2 (page 11)**
- **Appendix Figure 3 (page 11)**
- **Appendix Table 6 (page 12)**
- **Appendix Figure 4 (page 13)**
- **Appendix Table 7 (page 14 to 16)**
- **Appendix Figure 5 (page 17 to 18)**
- **Study group, Steering committee, Oversight boards, and Participating centres (page 20 to 21)**

Data linkage methods

Trial electronic case report forms (eCRFs) were collated on a six-weekly basis and trial clinics were advised to provide monthly GUMCAD surveillance returns as per established reporting streams. Data were linked with informed consent using clinic identifiers on the specified attendance date or up to a 30-day window. Trial participant tracking was possible at the enrolment clinic or across services where transfers were specified or where follow-up occurred in linked sexual health services sharing patient identifiers. Data quality was monitored through the duration of the trial period where unlinked eCRF records and/or service use queries (e.g. absence of HIV testing at enrolment) were identified to allow reconciliation at trial clinics. All reported HIV seroconversions were validated with trial clinics.

For final analyses, demographic and prescription information reported in eCRFs were prioritised in trial participants. Unlinked eCRF information was retained to allow availability of all PrEP-related provision and discontinuation information for analyses. Information from non-trial attendees derived from GUMCAD from respective trial clinic recruitment start (see Appendix Figure 1 and Appendix Table 1).

Appendix Figure 1: Trial data flow

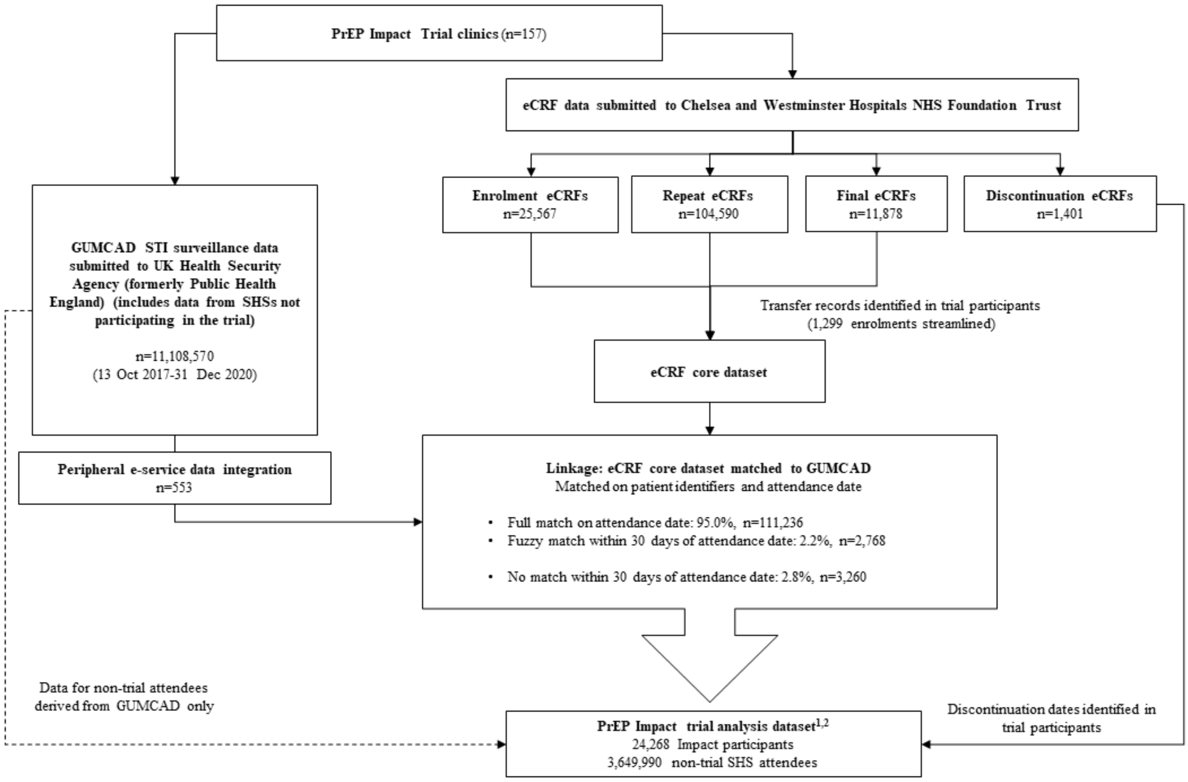

[1] With at least one SHS attendance from 13th October 2017 or respective trial clinic start date.

[2] Prior to further analysis restrictions - see Figure 1.

SHS=specialist sexual health services. eCRF=electronic case report form.

Appendix Table 1: Total records and data linkage in trial participant records from 13 October 2017 to 29 February 2020

	Total records[1]	Linkage to GUMCAD[2]		
		On-day (%)	Within 4-weeks (%)	No match (%)
**Enrolment eCRF[3]**	21,356	20,752 (97·2)	230 (1·1)	374 (1·8)
**Other eCRFs**	67,772	65,231 (96·3)	1,046 (1·5)	1,487 (2·2)
Repeat[4]	67,592	65,086 (96·3)	1,015 (1·5)	1,483 (2·2)
Discontinuation[4]	180	145 (80·6)	31 (17·2)	4 (2·2)
Final visit[5]	0	0	0	0
**Additional GUMCAD attendances[4]**	54,247	-	-	-
**E-service records[4]**	131	-	-	-

GUMCAD: GUMCAD STI surveillance system; eCRF: electronic case report form

[1]All trial participant eCRF and GUMCAD records, irrespective of eCRF match, were include in analysis datasets.

[2]Linked by patient identifiers and attendance date.

[3]One record per trial participant.

[4]May include multiple records per trial participant.

[5]Note, all final visits occurred after 29 February 2020.

Appendix Table 2: Description of population and denominators used for analyses

	Description	Exclusions	Total population	Relevant figures & tables	Notes
**1**	Descriptive characteristics of all Impact trial participants	Enrolled after 29th Feb 2020 n=2,912	21,356	Figure 1<br>Table 1	
**2**	Impact trial participants included in statistical analyses	Individuals with no evidence of ever receiving a prescription n=54<br><br>HIV positive at baseline n=10	21,292<br><br>MSM: 20,349<br>WP: 939	Figure 1<br>Table 2<br>Appendix Table 4	Denominator for the following analyses:<br>• Risk of HIV acquisition<br>• PrEP uptake<br>• PrEP coverage
**3**	HIV negative non-trial attendees included in statistical analyses	Sourcing PrEP elsewhere n=6,682	1,506,410<br><br>MSM: 144,921<br>WP: 1,324,261	Figure 1<br>Table 2<br>Appendix Table 3<br>Appendix Table 4	Denominator for the following analyses:<br>• Risk of HIV acquisition<br>• PrEP uptake<br>• PrEP coverage
**4**	Impact trial participants with at least one visit post-enrolment	One visit only n= 2,793	18,499<br><br>MSM: 17,770<br>WP: 728	Figure 1<br>Figure 2<br>Table 3<br>Table 4<br>Table 5<br>Appendix Table 4<br>Appendix Figure 2<br>Appendix Figure 3<br>Appendix Figure 4	Denominator for time-dependent analyses:<br>• Duration of HIV risk MSM only<br>• HIV and STI incidence MSM only<br>• Duration of PrEP use
**5**	HIV negative non-trial attendees with at least two visits	One visit only n= 926,165	580,245<br><br>MSM: 85,072<br>WP: 487,077	Figure 1<br>Figure 2<br>Table 3<br>Table 4<br>Table 5<br>Appendix Table 4<br>Appendix Figure 2<br>Appendix Figure 4	Denominator for time-dependent analyses:<br>• Duration of HIV risk MSM only<br>• HIV and STI incidence MSM only

MSM: Men who have sex with men; WP: women and other populations; PrEP: pre-exposure prophylaxis; STI: sexually transmitted infection

Appendix Table 3: Characteristics of HIV negative non-trial attendees accessing participating sexual health services between 13th October 2017 and 29th February 2020 by gender and sexual risk

	**All non-trial attendees**[1] n=1,506,410		**Cisgender MSM** n=144, 921		**Cisgender heterosexual men** n=562,219		**Cisgender women** n=761,787		**Transgender women** n=144		**Transgender men** n=111		**Cisgender men, unknown sexual orientation** n=26,098		**Unknown gender, unknown sexual orientation** n=11,130	
	n	%	n	%	n	%	n	%	n	%	n	%	n	%	n	%
Mean age (SD)	30·2 (10·5)		33·4 (12·1)		31·2 (10·9)		28·8 (9·6)		30·5 (10·5)		28·6 (9·6)		31·1 (11·1)		28·9 (9·5)	
Median age (IQR)	27 (22-35)		30 (24-40)		28 (23-36)		26 (22-33)		28 (23-34.5)		25 (22-33)		28 (23-36)		27 (22-33)	
Age range (years)	16-99		16-94		16-94		16-99		16-64		16-68		16-91		16-99	
**Age group**																
16-19	146,332	9·7	7,888	5·4	42,084	7·5	92,871	12·2	14	9·7	10	9·0	2,238	8·6	1,227	11·0
20-24	392,095	26·0	28,448	19·6	136,488	24·3	217,586	28·6	33	22·9	33	29·7	6,469	24·8	3,038	27·3
25-29	341,511	22·7	31,917	22·0	129,694	23·1	171,324	22·5	34	23·6	30	27·0	5,814	22·3	2,698	24·2
30-34	228,021	15·1	23,942	16·5	89,846	16·0	108,558	14·3	27	18·8	17	15·3	3,897	14·9	1,734	15·6
35-39	146,550	9·7	16,463	11·4	58,105	10·3	68,306	9·0	14	9·7	6	5·4	2,625	10·1	1,031	9·3
40-44	89,021	5·9	10,458	7·2	35,093	6·2	41,225	5·4	7	4·9	8	7·2	1,643	6·3	587	5·3
45-49	64,182	4·3	8,497	5·9	25,795	4·6	28,341	3·7	3	2·1	1	0·9	1,229	4·7	316	2·8
50-54	44,691	3·0	6,736	4·7	18,820	3·4	17,992	2·4	4	2·8	2	1·8	931	3·6	206	1·9
55-59	26,966	1·8	4,550	3·1	12,564	2·2	9,133	1·2	3	2·1	3	2·7	577	2·2	136	1·2
≥60	26,868	1·8	6,018	4·2	13,697	2·4	6,355	0·8	5	3·5	1	0·9	659	2·5	133	1·2
Unknown	173	0·0	4	0·0	33	0·0	96	0·0	0	0·0	0	0·0	16	0·1	24	0·2
**Ethnic group**																
White	1,047,938	69·6	108,536	74·9	381,287	67·8	538,740	70·7	108	75·0	83	74·8	14,085	54·0	5,099	45·8
Black African	72,056	4·8	2,153	1·5	33,719	6·0	34,531	4·5	0	0·0	2	1·8	1,287	4·9	364	3·3
Black Caribbean	54,210	3·6	2,260	1·6	22,522	4·0	27,949	3·7	5	3·5	4	3·6	1,253	4·8	217	2·0
Black other	16,324	1·1	733	0·5	7,095	1·3	8,088	1·1	4	2·8	3	2·7	328	1·3	73	0·7
Asian or Asian British	79,240	5·3	7,797	5·4	35,863	6·4	33,778	4·4	8	5·6	6	5·4	1,433	5·5	355	3·2
Mixed	71,727	4·8	5,965	4·1	26,160	4·7	38,027	5·0	11	7·6	4	3·6	1,241	4·8	319	2·9
Other	39,841	2·6	5,576	3·9	12,852	2·3	20,363	2·7	4	2·8	7	6·3	835	3·2	204	1·8
Unknown	125,074	8·3	11,901	8·2	42,721	7·6	60,311	7·9	4	2·8	2	1·8	5,636	21·6	4,499	40·4
**Region of birth**																
UK	1,016,318	67·5	90,823	62·7	397,209	70·7	512,763	67·3	86	59·7	81	73·0	10,855	41·6	4,501	40·4
Europe excluding UK	144,280	9·6	19,255	13·3	43,719	7·8	78,906	10·4	19	13·2	6	5·4	1,659	6·4	716	6·4
Caribbean	12,949	0·9	611	0·4	5,548	1·0	6,407	0·8	1	0·7	0	0·0	326	1·3	56	0·5

Sub-Saharan Africa	59,766	4·0	2,738	1·9	27,563	4·9	28,150	3·7	1	0·7	2	1·8	1,061	4·1	251	2·3
South Asia	22,174	1·5	2,172	1·5	11,680	2·1	7,889	1·0	1	0·7	0	0·0	340	1·3	92	0·8
Central America	1,406	0·1	358	0·3	382	0·1	635	0·1	0	0·0	0	0·0	21	0·1	10	0·1
North America	10,735	0·7	1,882	1·3	2,895	0·5	5,733	0·8	3	2·1	1	0·9	161	0·6	60	0·5
South America	15,097	1·0	3,046	2·1	3,482	0·6	8,166	1·1	16	11·1	8	7·2	316	1·2	63	0·6
Other	53,998	3·6	7,859	5·4	20,136	3·6	24,892	3·3	9	6·3	9	8·1	868	3·3	225	2·0
Unknown	169,687	11·3	16,177	11·2	49,605	8·8	88,246	11·6	8	5·6	4	3·6	10,491	40·2	5,156	46·3
**Region of residence[2]**																
London	428,635	28·5	57,338	39·6	149,156	26·5	210,137	27·6	50	34·7	18	16·2	8,703	33·4	3,233	29·1
Midlands and East	385,777	25·6	28,774	19·9	146,658	26·1	199,993	26·3	14	9·7	23	20·7	9,235	35·4	1,080	9·7
North	320,808	21·3	28,182	19·5	122,931	21·9	161,204	21·2	20	13·9	18	16·2	4,837	18·5	3,616	32·5
South	337,047	22·4	26,600	18·4	129,578	23·1	175,632	23·1	60	41·7	52	46·9	2,445	9·4	2,680	24·1
UK other	12,873	0·9	1,554	1·1	5,422	1·0	5,678	0·8	0	0·0	0	0·0	122	0·5	97	0·9
Abroad	1,278	0·1	205	0·1	540	0·1	507	0·1	0	0·0	0	0·0	15	0·1	11	0·1
Unknown	19,992	1·3	2,268	1·6	7,934	1·4	8,636	1·1	0	0·0	0	0·0	741	2·8	413	3·7
**Index of Multiple Deprivation (IMD)[3]**																
1 most deprived	349,662	23·2	29,939	20·7	129,730	23·1	180,517	23·7	34	23·6	26	23·4	6,930	26·6	2,486	22·3
2	381,461	25·3	41,493	28·6	135,349	24·1	193,816	25·4	45	31·3	33	29·7	7,381	28·3	3,344	30·0
3	298,909	19·8	30,003	20·7	110,287	19·6	151,340	19·9	22	15·3	27	24·3	5,301	20·3	1,929	17·3
4	241,244	16·0	22,590	15·6	92,926	16·5	120,991	15·9	30	20·8	20	18·0	3,186	12·2	1,501	13·5
5 least deprived	201,002	13·3	16,871	11·6	80,032	14·2	100,310	13·2	13	9·0	5	4·5	2,422	9·3	1,349	12·1
Unknown	34,132	2·3	4,025	2·8	13,895	2·5	14,813	1·9	0	0·0	0	0·0	878	3·4	521	4·7

MSM: Men who have sex with men; UK: United Kingdom

[1] Based on characteristics reported at follow-up start date. HIV negative non-trial attendees accessing a participating sexual health service at least once after recruitment at that SHS had begun and before the end of February 2020, with a recorded HIV test and no evidence of obtaining PrEP from another source.

[2] Region of residence based on lower layer super output area (LSOA).

[3] Index of multiple deprivation (IMD) quintiles based on residence LSOA.

SD=standard deviation. IQR=interquartile range.

Appendix Table 4: MSM trial participants and non-trial attendees at risk of HIV acquisition

MSM attendees	All attendees[1] n=165,270		Included in time-dependent analyses[2] n=102,842	
	n	%	n	%
**Trial participants all**	20,349	100·0	17,770	100·0
Ever at risk of HIV acquisition	20,349	100·0	17,770	100·0
With PrEP eligibility coding	20,349	100·0	17,770	100·0
With additional MoHR	17,680	86·9	16,790	94·5
No PrEP eligibility coding or additional MoHR	0	0·0	0	0·0
**Non-trial attendees all**	144,921	100·0	85,072	100·0
Ever at risk of HIV acquisition	73,930	51·0	61,324	72·1
With PrEP eligibility coding	14,531	10·0	11,541	13·6
With additional MoHR	68,998	47·6	58,796	69·1
No PrEP eligibility coding or additional MoHR	70,991	49·0	23,748	27·9

MSM: Men who have sex with men; PrEP: pre-exposure prophylaxis; MoHR: markers of higher risk

[1] HIV negative attendees accessing a participating sexual health service (SHS) at least once after recruitment at that SHS had begun and before the end of February 2020, with a recorded HIV test and no evidence of obtaining PrEP from another source; excludes 54 trial participants with no record of receiving a PrEP prescription during the analysis period, and ten trial participants who likely acquired HIV prior to enrolment and use of trial PrEP.

[2] HIV negative attendees accessing a participating sexual health service (SHS) with at least one follow-up attendance after enrolment (Impact participants) or at least two visits after recruitment at that SHS had started (non-trial attendees) to the end of February 2020, with a recorded HIV test and no evidence of obtaining PrEP from another source; excludes 54 trial participants with no record of receiving a PrEP prescription during the analysis period, ten trial participants who likely acquired HIV prior to enrolment and use of trial PrEP, and 926,958 individuals with only one SHS attendance (2,793 trial participants; 926,165 non-trial attendees).

Ever at risk of HIV acquisition = PrEP eligibility, offer or prescription codes, or markers of higher risk (MoHR) recorded follow-up start to end of February 2020.

Appendix Table 5: Sexual health service attendees[1] included in time-dependent analyses

	Attendees included in time-dependent analyses				MSM attendees included in time-dependent analyses					
	Impact trial participants		Non-trial attendees (all)		MSM Impact trial participants		MSM non-trial attendees (all)		MSM non-trial attendees at risk of HIV acquisition	
	n=18,499	%	n=580,245	%	n=17,770	%	n=85,072	%	n=61,324	%
Mean age (SD)	35·6 (10·9)		29·7 (10·5)		35·6 (10·8)		33·3 (11·9)		33·2 (11·7)	
Median age (IQR)	33 (27-42)		27 (22-34)		33 (27-42)		30 (25-39)		30 (25-39)	
Age range (years)	16-86		16-93		16-86		16-90		16-89	
**Age group**										
16-19	391	2·1	64,200	11·1	378	2·1	4,659	5·5	3,198	5·2
20-24	2,118	11·5	157,944	27·2	2,007	11·3	16,545	19·5	11,859	19·3
25-29	3,877	21·0	131,707	22·7	3,729	21·0	19,020	22·4	14,085	23·0
30-34	3,547	19·2	83,977	14·5	3,411	19·2	14,205	16·7	10,437	17·0
35-39	2,794	15·1	52,495	9·1	2,706	15·2	9,885	11·6	7,243	11·8
40-44	1,882	10·2	31,535	5·4	1,807	10·2	6,236	7·3	4,455	7·3
45-49	1,604	8·7	22,332	3·9	1,548	8·7	4,776	5·6	3,361	5·5
50-54	1,134	6·1	15,623	2·7	1,092	6·2	3,747	4·4	2,609	4·3
55-59	604	3·3	9,573	1·7	578	3·3	2,531	3·0	1,758	2·9
≥60	548	3·0	10,851	1·9	514	2·9	3,468	4·1	2,319	3·8
Unknown	0	0.0	8	0·0	0	0.0	0	0.0	0	0.0
**Region of birth**										
UK	11,218	60·6	395,628	68·2	10,833	61·0	53,729	63·2	38,031	62·0
Europe excluding UK	2,868	15·5	59,600	10·3	2,795	15·7	12,131	14·3	9,267	15·1
Caribbean	87	0·5	5630	1·0	85	0·5	395	0·5	297	0·5
Sub-Saharan Africa	510	2·8	23893	4·1	457	2·6	1,722	2·0	1287	2·1
South Asia	235	1·3	8117	1·4	227	1·3	1,386	1·6	1010	1·7
Central America	49	0·3	582	0·1	46	0·3	208	0·2	161	0·3
North America	327	1·8	4064	0·7	318	1·8	1,011	1·2	726	1·2
South America	646	3·5	6724	1·2	589	3·3	1,936	2·3	1508	2·5
Other	1,192	6·4	21,891	3·8	1,138	6·4	4,682	5·5	3,531	5·8
Unknown	1,367	7·4	54,116	9·3	1,282	7·2	7,872	9·3	5,506	9·0
**STI in past year**										
No previous STI, no STI diagnosis at enrolment	8,709	47·1	95,486	16·5	8,275	46·6	15,003	17·6	14,034	22·9

Unknown previous STI, no STI diagnosis at enrolment	1,988	10·8	373,302	64·3	1,878	10·6	48,489	57·0	30,533	49·8
Previous STI and/or STI diagnosis at enrolment	7,802	42·2	111,457	19·2	7,617	42·9	21,580	25·4	16,757	27·3
**Index of Multiple Deprivation (IMD)[2]**										
1 most deprived	3,818	20·6	143,811	24·8	3,641	20·5	18,239	21·4	13,104	21·4
2	6,038	32·6	152,586	26·3	5,830	32·8	24,808	29·2	18,220	29·7
3	3,992	21·6	114,002	19·7	3,833	21·6	17,726	20·8	12,895	21·0
4	2,650	14·3	89,192	15·4	2,548	14·3	13,115	15·4	9,347	15·2
5 least deprived	1,762	9·5	71,640	12·4	1,698	9·6	9,548	11·2	6,699	10·9
Unknown	239	1·3	9,014	1·6	220	1·2	1,636	1·9	1,059	1·7
**Region of residence**										
London	10,052	54·3	175,453	30·2	9,685	54·5	34,270	40·3	25,936	42·3
Midlands and East	2,289	12·4	143,067	24·7	2,187	12·3	16,518	19·4	11,403	18·6
North	2,816	15·2	126,973	21·9	2,724	15·3	16,865	19·8	11,801	19·2
South	3,101	16·8	125,727	21·7	2,952	16·6	15,781	18·6	11,124	18·1
UK other	69	0·4	4,440	0·8	65	0·4	737	0·9	485	0·8
Abroad	13	0·1	398	0·1	13	0·1	94	0·1	63	0·1
Unknown	159	0·9	4,187	0·7	144	0·8	807	1·0	512	0·8
**Ethnic group**										
White	14,093	76·2	404,520	69·7	13,664	76·9	64,293	75·6	46,117	75·2
Black African	323	1·8	30,193	5·2	269	1·5	1,359	1·6	1,049	1·7
Black Caribbean	299	1·6	25,183	4·3	286	1·6	1,499	1·8	1,199	2·0
Black other	114	0·6	7,096	1·2	107	0·6	460	0·5	349	0·6
Asian or Asian British	934	5·1	29,513	5·1	874	4·9	4,812	5·7	3,666	6·0
Mixed	789	4·3	30,956	5·3	734	4·1	3,636	4·3	2,658	4·3
Other	727	3·9	16,305	2·8	688	3·9	3,398	4·0	2,511	4·1
Unknown	1,220	6·6	36,479	6·3	1,148	6·5	5,615	6·6	3,775	6·2
**Regimen at enrolment[3]**										
Daily	15,200	82·2			14,589	82·1				
Event Based Dosing	2,604	14·1			2,521	14·2				
Unknown	695	3·8			660	3·7				

MSM: Men who have sex with men; UK: United Kingdom; STI: Sexually transmitted infection; EBD: event-based dosing.

[1] HIV negative attendees accessing a participating sexual health service (SHS) with at least one follow-up attendance after enrolment (Impact participants) or at least two visits after recruitment at that SHS had started (non-trial attendees) to the end of February 2020, with a recorded HIV test and no evidence of obtaining PrEP from another source; excludes 54 trial participants with no record of receiving a PrEP prescription during the analysis period, ten trial participants who likely acquired HIV prior to enrolment and use of trial PrEP, and 926,958 individuals with only one SHS attendance (2,793 trial participants; 926,165 non-trial attendees).

[2] Index of multiple deprivation (IMD) quintiles based on residence lower layer super output area (LSOA).

[3] Regimen based on coding reported on enrolment date, or, where not reported on enrolment date, coding reported in first subsequent visit where reported.

SD=standard deviation. IQR=interquartile range.

Appendix Figure 2: Proportion of follow-up time at risk of HIV acquisition among MSM who were in a period of risk for HIV during part of their follow-up

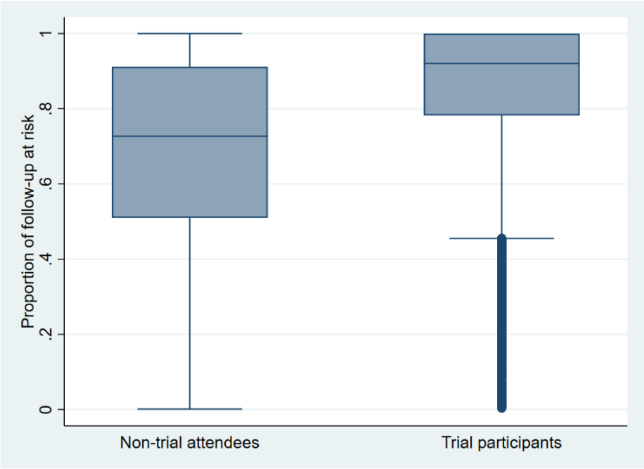

Outliers that differ significantly from the rest of the dataset and fall outside the boundary of the whiskers are plotted as individual data points beyond the whiskers.

Appendix Figure 3: Proportion of follow-up time in all Impact trial participants with enough pills to protect against HIV acquisition

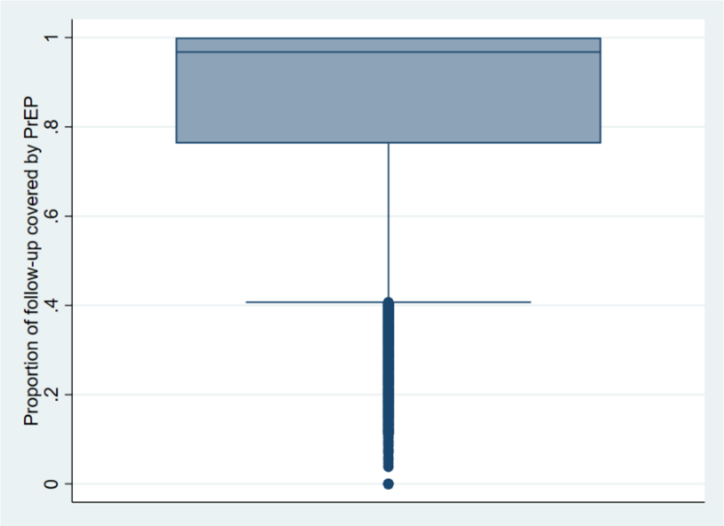

Outliers that differ significantly from the rest of the dataset and fall outside the boundary of the whiskers are plotted as individual data points beyond the whiskers.

Appendix Table 6: PrEP need in MSM

PrEP need	n	%
Impact participants	20,349	
Non-trial attendees ever at risk of HIV acquistion[1]	73,541	
Non-trial attendees who seroconverted[2]	587	
Non-trial attendees sourcing PrEP elsewhere	6,323	
**Total estimated PrEP need**	**100,800 (42,367 per annum)**	
**MSM attendees[3]**	**165,270**	
**Impact participants (all)**	20,349	100·0
Ever at risk of HIV acquisition	20,349	100·0
With PrEP eligibility coding	20,349	100·0
With additional MoHR	17,680	86·9
No PrEP eligibility coding or additional MoHR	0	0·0
**Non-trial attendees (all)**	144,921	100·0
Ever at risk of HIV acquisition	73,930	51·0
With PrEP eligibility coding	14,531	10·0
With additional MoHR	68,998	47·6
No PrEP eligibility coding or additional MoHR	70,991	49·0
**MSM seroconversions**	**611**	
**Impact participants who seroconverted[4]**	24	100·0
Ever at risk of HIV acquisition	24	100·0
With PrEP eligibility coding	24	100·0
With additional MoHR	22	91·7
No PrEP eligibility coding or additional MoHR	0	0·0
**Non-trial attendees who seroconverted**	587	100·0
Ever at risk of HIV acquisition	389	66·3
With PrEP eligibility coding	52	8·9
With additional MoHR	378	64·4
No PrEP eligibility coding or additional MoHR	198	33·7
**MSM sourcing PrEP elsewhere**	**6,323**	

PrEP: pre-exposure prophylaxis; MSM: Men who have sex with men; MoHR: Markers of higher risk;

[1] Excludes 389 non-trial attendees ever at risk of HIV acquisition who seroconverted (see 'Non-trial attendees who seroconverted').

[2] Includes 389 non-trial attendees ever at risk of HIV acquisition and 198 non-trial attendees without eligibility or markers of higher risk (MoHR).

[3] HIV negative attendees accessing a participating sexual health service (SHS) at least once after recruitment at that SHS had begun and before the end of February 2020, with a recorded HIV test and no evidence of obtaining PrEP from another source; excludes 54 trial participants with no record of receiving a PrEP prescription during the analysis period, and ten trial participants who likely acquired HIV prior to enrolment and use of trial PrEP.

4 Includes 24 of 26 MSM with a new HIV diagnosis during the analysis period; exclusions include one individual who did not have a second visit post-enrolment and one individual with follow-up ending at their last SHS visit, which occurred prior to HIV diagnosis.

Ever at risk of HIV acquisition: PrEP eligibility, offer or prescription codes, or markers of higher risk (MoHR) from follow-up start to end of February 2020

Appendix Figure 4. Distribution of MSM trial participants and STI diagnoses by number of diagnoses per participant during follow up

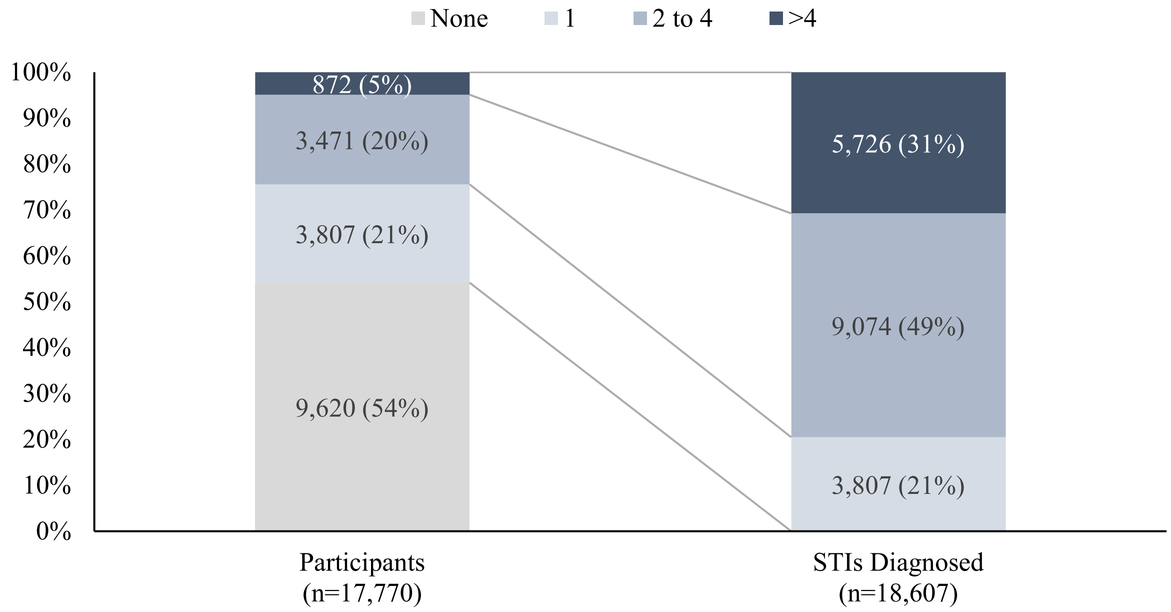

HIV negative attendees accessing a participating sexual health service (SHS) with at least one follow-up attendance after enrolment to the end of February 2020; excludes 54 trial participants with no record of receiving a PrEP prescription during the analysis period, ten trial participants who likely acquired HIV prior to enrolment and use of trial PrEP, and trial participants with only one SHS attendance

Appendix Table 7: Definitions of measures used in analyses (cross-reference with Appendix Tables 2, 5)

Measure	Metric	Numerator	Denominator	Notes
Risk of HIV acquisition	Count	Number of SHS attendees clinically assessed as eligible for PrEP during the analysis period (i.e., with reported PrEP eligibility, offer or prescription codes) and/or clinical and behavioural markers known to be associated with higher risk of acquiring HIV (markers of higher risk, MoHR) that were reported for visits in the 12 months prior to, and during the analysis period for HIV incidence.<br><br>MoHR include: a rectal bacterial STI diagnosis, use of HIV post-exposure prophylaxis, being a sex worker, being in contact of someone diagnosed with HIV or syphilis (attending as a consequence of partner notification)	N/A	Calculated for MSM only
HIV incidence	Rate (per 100 person years)	HIV diagnosis (i.e., first positive HIV test)	Total follow-up time defined below:<br><br>Trial participants: date of enrolment until first date of a positive HIV test or censoring (discontinuation from trial or at the last visit before the end of February 2020)<br><br>Non-trial participants: first visit after recruitment of participants had started at that site until first	Calculated for MSM included in time-dependent analyses (see Appendix Table 2, Appendix Table 5)<br><br>Main analysis includes all MSM; sensitivity analysis includes MSM at risk of HIV acquisition<br><br>See measure ‘Risk of HIV acquisition’

			date of a positive HIV test or censoring (last visit before the end of February 2020)	
Bacterial STI incidence	Rate (per 100 person years)	Chlamydia diagnosis (i.e., positive chlamydia test)<br><br>Gonorrhoea diagnosis (i.e., positive gonorrhoea test)<br><br>Syphilis diagnosis (i.e., positive syphilis test)<br><br>For any STI diagnosis:<br>A chlamydia, gonorrhoea, and/or syphilis diagnosis	Total follow-up time defined below:<br><br>Trial participants: date of enrolment until first date of a positive HIV test or censoring (discontinuation from trial or at the last visit before the end of February 2020)<br><br>Non-trial participants: first visit after recruitment of participants had started at that site until first date of a positive HIV test or censoring (last visit before the end of February 2020)	Calculated for MSM included in time-dependent analyses (see Appendix Table 2, Appendix Table 5)<br><br>Unadjusted and adjusted for the number of STI tests recorded during follow-up<br><br>Concurrent infections of different bacterial STIs were counted separately when estimating the incidence of any bacterial STI.<br><br>For individual STI incidence estimates, positive results of the same infection at multiple anatomical sites on the same day (e.g., pharyngeal and rectal gonorrhoea) were considered as a single infection, while concurrent diagnoses of different infections at the same or different anatomical sites were considered separate infections.<br><br>Tests for different bacterial STIs were counted separately, while tests for the same infection at different anatomical sites were counted as a single test.
Duration of PrEP use	Proportion	Total number of PrEP pills prescribed	Total follow-up time defined below:	Calculated for MSM included in time-dependent analyses (see

			Trial participants: date of enrolment until first date of a positive HIV test or censoring (discontinuation from trial or at the last visit before the end of February 2020)<br><br>Non-trial participants: first visit after recruitment of participants had started at that site until first date of a positive HIV test or censoring (last visit before the end of February 2020)	Appendix Table 2, Appendix Table 5)
Duration of HIV risk	Proportion	The interval between two attendances where at the most recent attendance the person was categorised as at risk of HIV acquisition	Total follow-up time defined below:<br><br>Trial participants: date of enrolment until first date of a positive HIV test or censoring (discontinuation from trial or at the last visit before the end of February 2020)<br><br>Non-trial participants: first visit after recruitment of participants had started at that site until first date of a positive HIV test or censoring (last visit before the end of February 2020)	Calculated for MSM included in time-dependent analyses (see Appendix Table 2, Appendix Table 5)<br><br>Maximum duration of 6 months was considered if interval between two attendances exceeded 6 months<br><br>See measure 'Risk of HIV acquisition'
Population need	Count	Combined count of: all trial participants, non-trial attendees categorised at risk of HIV acquisition, non-trial attendees who were not categorised at risk of HIV acquisition but who sero-converted during follow-up, those obtaining PrEP at another source	N/A	See measure 'Risk of HIV acquisition'

PrEP uptake	Proportion	Number of SHS attendees clinically assessed as eligible for PrEP during the analysis period (i.e., with reported PrEP eligibility, offer or prescription codes), including at times when enrolment was paused, who began PrEP through participation in the trial	Number of SHS attendees clinically assessed as eligible for PrEP during the analysis period (i.e., with reported PrEP eligibility, offer or prescription codes)	
PrEP coverage	Proportion	Number of SHS attendees clinically assessed as eligible for PrEP during the analysis period (i.e., with reported PrEP eligibility, offer or prescription codes), including at times when enrolment was paused, who began PrEP through participation in the trial	Number of SHS attendees ever categorised at risk of HIV acquisition during the analysis period, whether recognised clinically and coded or not	Calculated for MSM only<br><br>See measure 'Risk of HIV acquisition'

MSM= men who have sex with men

SHS= specialist sexual health services

MoHR: markers of higher risk of HIV acquisition which include the report of a rectal bacterial STI diagnosis, use of HIV post-exposure prophylaxis, being a sex worker, being in contact of someone diagnosed with HIV or syphilis (attending as a consequence of partner notification) in the 12 months prior to, and during the analysis period

Appendix Figure 5. Trends in HIV and STI incidence by follow-up term in trial participants and non-trial attendees

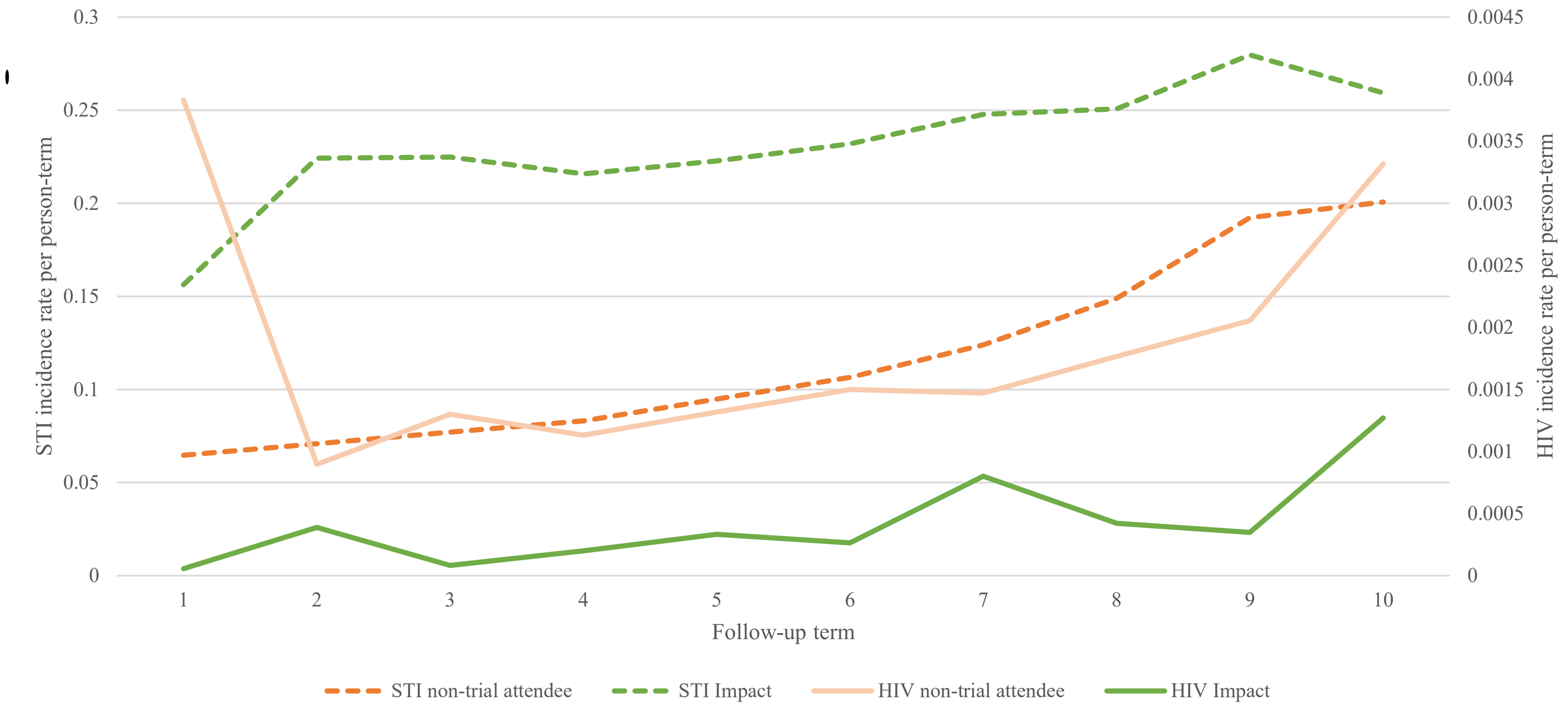

A follow-up term signifies time from enrolment (Impact participants) or first follow-up attendance (non-trial participants) and comprises a 90-day period (e.g., Term 1 represents the first 90 days from enrolment/first follow-up). Persons are assumed contribute to follow-up time from first to last term appearance (e.g., a person appearing in Term 1 and Term 5 will be counted in Term 1, Term 2, Term 3, Term 4, and Term 5).

	Follow-up term									
	1	2	3	4	5	6	7	8	9	10
Non-trial attendees										
Number of persons	85,072	57,977	46,131	35,376	27,315	19,989	13,600	7,928	3,407	603
Number of STIs	5,501	4,111	3,554	2,939	2,590	2,130	1,685	1,181	655	121
STI incidence rate	0·0647	0·0709	0·0770	0·0831	0·0948	0·1066	0·1239	0·1490	0·1923	0·2007
Number of HIV seroconversions	326	52	60	40	36	30	20	14	7	2
HIV incidence rate	0·0038	0·0009	0·0013	0·0011	0·0013	0·0015	0·0015	0·0018	0·0021	0·0033
Impact participants										
Number of persons	17,770	15,408	12,116	9,996	8,999	7,554	6,238	4,744	2,864	787
Number of STIs	2,777	3,454	2,723	2,157	2,005	1,752	1,545	1,189	801	204
STI incidence rate	0·1563	0·2242	0·2247	0·2158	0·2228	0·2319	0·2477	0·2506	0·2797	0·2592
Number of HIV seroconversions	1	6	1	2	3	2	5	2	1	1
HIV incidence rate	0·0001	0·0004	0·0001	0·0002	0·0003	0·0003	0·0008	0·0004	0·0003	0·0013

Study Group

The Impact Study Group is composed of the authors of this manuscript and:

Yusef Azad MD[1], Pallavi Chhibbar MSc[2], Flavien Coukan MSc[3], Matthew Hibbert PhD[3], Emily L Mason MSc[3], Anthony Nardone PhD[3], Ola Oladela BSc[2], Roeann Osman MSc[3], Branca Pereira PhD[2], Hannah Reaney MSc[2], Beverley White-Alao BSc[2]

[1]National AIDS Trust, London, United Kingdom

[2]Chelsea and Westminster Hospital NHS Foundation Trust, London, UK

[3]UK Health Security Agency, London, UK

Trial Steering Committee: C Estcourt (chair), Y Azad (to - 08/19) D Gold (from – 09/19), D Goldberg, T Peto, A Rodger, C Sabin

Community Advisory Board: Y Azad (co-chair to – 8/19), D Gold (co-chair from – 9/19), T Nardone (co-chair to – 06/2018), A Sullivan (co-chair from- 07/2018), Africa Advocacy Foundation (J Akello, D Onyango,E Phiri), African Equality Foundation (E Sesange), African Eye Trust (D Ravenor), African Health Policy Network (M Ndawana), CliniQ (M Ross), CNWL and SurvivorsUK (D Campbell), LGBT Foundation (L Duffy), LGBT Foundation (M Petch), NAM (G Cairns), NAM, POB Lay Representative (R Pebody), POB Lay Representative (F Labwo), Porn for PrEP UK, National Ugly Mugs (J Domino), PrEPster (W Nutland, P Samba), Sophia Forum (J Stevenson, S Strachan), Spectra and GMI Partnership (K Skipper), Terrence Higgins Trust (I Green), THT, PrEPster (M Thompson), UK Community Advisory Board (P Clift), Yorkshire MESMAC (T Doyle), UKHSA (A Winter), NHS England (F Woodward)

Programme Oversight Board: John Stewart (chair from 4/17, NHSE), Nick Phin (chair 4/17-3/19, UKHSA), Geoff Dusheiko (chair, 3/19-12/19, UKHSA), Gwenda Hughes (co-chair from 01/2020, UKHSA), Claire Foreman, NHS England; Andre Charlett, Kevin Fenton, Bernie Hannigan, UK Health Security Agency (form. Public Health England); Paul Ogden, Local Government Association; Robbie Currie, Local Authority Commissioning Representative; Louise Smith, Association of Directors of Public Health representative, Janet Wilson, Leeds Teaching Hospitals NHS Trust; Isabel Carrick, East Riding of Yorkshire Council; Liz Rodrigo; Leicester City Council; Rob Cookson, Roger Pebody, Florence Jane Labwo Oree, lay representatives

Sponsor: D Foster, E Ramhamadany, M Johnson, Chelsea and Westminster NHS Foundation Trust

NHS England: M Pryor

UKHSA: H Mohamed, R Harris, N Field, V Hall, N Iyanger

Participating centres: Barking Hospital (A Umaipalan), Barts Health NHS Trust (A Williams), Berkshire Healthcare NHS Foundation Trust (N Pal), Blackpool Teaching Hospitals NHS Foundation Trust (W Wasef), Bolton NHS Foundation Trust (E Morgan), Brook (AS Menon-Johansson), Buckinghamshire Healthcare NHS Trust (R Malek), Calderdale and Huddersfield NHS Foundation Trust (L Short), Cambridgeshire Community Services NHS Trust, iCaSH (N David), Central and North West London NHS Foundation Trust (R Gilson), Central London Community Healthcare NHS Trust (A Samarawickrama), Chelsea and Westminster Hospital NHS Foundation Trust, 10 Hammersmith Broadway (M Rayment), Chelsea and Westminster Hospital NHS Foundation Trust, 56 Dean Street (A McOwan), Chelsea and Westminster Hospital NHS Foundation Trust, John Hunter Clinic (G Jagjit Singh), Chelsea and Westminster Hospital NHS Foundation Trust, West Middlesex University Hospital (S de Silva), City Healthcare Partnership, Hull (H McClean), County Durham and Darlington NHS Foundation Trust (A Wardropper), Coventry & Warwickshire Partnership NHS Trust (H Taha), Croydon Sexual Health (D Phillips), Derbyshire Community Health Services NHS Foundation Trust (F Nathani), Devon Sexual Health, Exeter (J Shaw), Devon Sexual Health, Torbay (N Khatib), Doncaster and Bassetlaw Teaching Hospitals NHS Foundation Trust (C Ryan), Dorset County Hospital NHS Foundation Trust (S Scofield), East Suffolk and North East Essex NHS Foundation Trust (M Ramogi), East Sussex Healthcare NHS Trust (K Aderogba), Epsom and St Helier University Hospitals NHS Foundation Trust (O Davies), George Eliot Hospital NHS Trust (D Natin), Gloucestershire Health and Care Services NHS Trust (A De Burgh-Thomas), Great Western Hospitals NHS Foundation Trust (S Forsyth), Guy's and St Thomas' NHS Foundation Trust (A Nori), Herefordshire and Worcestershire Health and Care NHS Trust (S Bhaduri), Homerton Healthcare NHS Foundation Trust (I Reeves), Imperial College Healthcare NHS Trust, St Mary's Hospital (O Dosekun), Kent Community Health NHS Foundation Trust (MY Tung), King's College Hospital NHS Foundation Trust (M Brady), Leeds Teaching Hospitals NHS Trust (A Evans), Lewisham and Greenwich NHS Trust (S Kegg), Lincolnshire Community Health Services NHS Trust (S Wellwood), Liverpool University Hospitals NHS Foundation Trust, Axess Sexual Health Halton and Warrington (D Mandal), Liverpool University Hospitals NHS Foundation Trust, Axess Sexual Health Liverpool (M Lawton), Liverpool University Hospitals NHS Foundation Trust, Macclesfield District General Hospital (NB Connolly), Locala CIC (VR Nair), Locala Sexual Health, Bradford (N Fearnley), Locala Sexual Health, Dewsbury (A Mammen-Tobin), London North West University Healthcare NHS Trust (J McSorley), Luton Sexual Health (T Balachandran), Maidstone and Tunbridge Wells NHS Trust, Rubin Clinic, Maidstone Hospital (L Navaratne), Manchester University NHS Foundation Trust (C Ward), Manchester University NHS Foundation Trust (A Ng), Midlands Partnership NHS Foundation Trust, Cobridge Sexual Health Centre (L Goodall), Midlands Partnership NHS Foundation Trust, Haymarket Health (V Kumar), Milton Keynes University Hospital NHS Foundation Trust (P Williams), Newcastle upon Tyne Hospitals NHS Foundation Trust, New Croft Clinic (S Duncan), North Cumbria Integrated Care NHS Foundation Trust (M Phillips), North Manchester General Hospital, Manchester University NHS Foundation Trust (A Tomkins), North Middlesex University Hospital NHS Foundation Trust (WC Loke), Northamptonshire Integrated Sexual Health and HIV (S Herbert), Northern Devon Healthcare NHS Trust (E Claydon), Northumbria Healthcare NHS Foundation Trust (O Hotonu), Nottingham University Hospitals NHS Trust (A Fox), Oxfordshire Sexual Health Service (NH Fadzillah), Royal Berkshire NHS Foundation Trust (D Ellis), Royal Wolverhampton NHS Trust (A Tariq), Salisbury NHS Foundation Trust (G Morris), Sheffield Teaching Hospitals NHS Foundation Trust (K Rogstad), Sherwood Forest Hospitals NHS Foundation Trust, King's Mill Hospital (E Carlin), Solent NHS Foundation Trust (A Blume), Somerset NHS Foundation Trust (S Cherif), South Tyneside and Sunderland NHS Foundation Trust, Sunderland Royal Hospital (S Bushby), South Tyneside and Sunderland NHS Foundation Trust, Sunderland Royal Hospital (M Basta), Spectrum Community Health CIC (NMJ Wright), St Helens and Knowsley Hospitals (E Acha), The Dudley Group NHS Foundation Trust (A El-Dalil), The Rotherham NHS Foundation Trust (N Gupta), The Wolverton Centre, Kingston Hospital NHS Foundation Trust (B Nathan), University Hospitals Birmingham NHS Foundation Trust (D White), University Hospitals Bristol and Weston NHS Foundation Trust (S Stockwell), University Hospitals Dorset NHS Foundation Trust (B Herieka), University Hospitals Plymouth NHS Trust (Z Warwick), University Hospitals Sussex NHS Foundation Trust (D Richardson), Virgin Care Services Ltd, Fountains Health (J Evans-Jones), Virgin Care Services Ltd, Northern Lincolnshire Integrated Sexual Health Service (P Gupta), Virgin Care Services Ltd, Northern Lincolnshire Integrated Sexual Health Service (U Joshi), Virgin Care Services Ltd, Oldham Integrated Care Centre (F Siddiqui), Virgin Care Services Ltd, Teesside Sexual Health (S Tayal), Walsall Healthcare NHS Trust (J Arumainayagam), Western Sussex Sexual Health Service (J Zhou), Wirral Community Health and Care NHS Foundation Trust (M Wood), Worcestershire Health and Care NHS Trust (S Bhaduri), York Teaching Hospitals NHS Foundation Trust (I Fairley)
